# Microbial Signals in Cancer: Dissecting Host–Microbiota–Tumor Interactions and Potential Therapeutic Strategy

**DOI:** 10.1002/mco2.70994

**Published:** 2026-09-11

**Authors:** Juan Lu, Mengjuan Xuan, Liang Yu, Zhongyang Xie, Qingfei Chu, Shihui Wei, Tuokai Wang, Chen Xue, Lanjuan Li

**Affiliations:** ^1^ State Key Laboratory For Diagnosis and Treatment of Infectious Diseases National Clinical Research Center for Infectious Diseases Collaborative Innovation Center For Diagnosis and Treatment of Infectious Diseases China‐Singapore Belt and Road Joint Laboratory on Infection Research and Drug Development National Medical Center For Infectious Diseases The First Affiliated Hospital Zhejiang University School of Medicine Hangzhou China

**Keywords:** bacteriophage, cancer, engineering bacteria, immunotherapy, microbiota

## Abstract

Cancers develop within a host ecosystem in which kinds of factors such as gut and tumor‐resident microbiota influence the tumor microenvironment (TME) and therapeutic response. High‐throughput sequencing has revealed the presence of low‐biomass bacteria, fungi, and viruses across diverse malignancies, establishing the intratumoral microbiota as a fundamental TME component. However, evidence remains fragmented across descriptive associations, unclear mechanisms, and early clinical interventions, limiting casual interpretation and clinical translation. This review dissects the host–microbiota–tumor axis from intratumoral origins and colonization to pattern‐recognition signaling, including TLR–NF‐κB and cGAS–STING, and oncogenic networks such as Wnt/β‐catenin, JAK–STAT, and PI3K–AKT. Moreover, it also discusses how microbial metabolites like short‐chain fatty acids, secondary bile acids, and tryptophan derivatives reshape immune cell phenotypes and tumor cell metabolism. We then synthesize evidence linking microbiota to immune checkpoint blockade, chemotherapy resistance, radiotherapy toxicity, diagnosis, and prognosis. Finally, we compare translational strategies, including fecal microbiota transplantation (FMT), engineered bacteria, oncolytic viruses, and bacteriophages, together with their safety and standardization barriers. By connecting molecular mechanisms with preclinical and clinical evidence, this review comprehensively provides a framework for causal, biomarker‐guided microbiota interventions.

## Graphical abstract

Microbial signals from gut and tumor‐resident bacteria, fungi, viruses, and bacteriophages reshape immune and metabolic networks within the tumor microenvironment, thereby influencing cancer progression, treatment response. Therapeutic modulations, including dietary interventions, probiotics, antibiotics, fecal microbiota transplantation, engineered bacteria, oncolytic viruses, and bacteriophages, profoundly influence the efficacy of clinical treatments such as immunotherapy, chemotherapy, and radiotherapy.

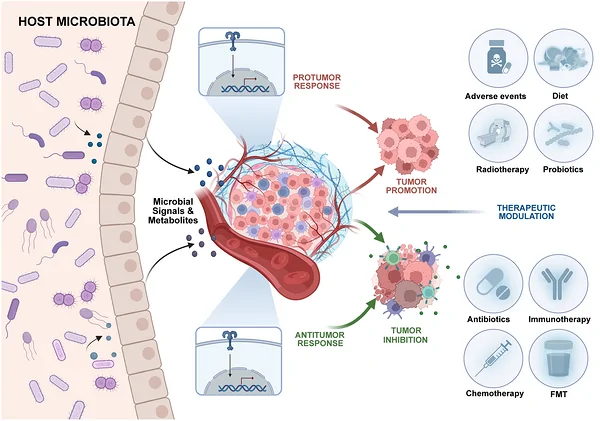

## 1 | Introduction

The human body is not merely an isolated biological entity but a superorganism consisting of trillions of human cells and an even more extensive symbiotic microbial community [1, 2]. These microorganisms play an essential role in critical processes such as immune regulation, metabolic balance, and barrier defense, thus maintaining physiological homeostasis [3, 4, 5]. The connection between microorganisms and disease dates back to ancient Egypt, where primitive therapies using infection to induce tumor regression were recorded [6]. In the late 19th century, Busch and Coley observed tumor reduction in certain cancer patients following severe bacterial infections, laying the groundwork for early microbial‐based immunotherapy [7, 8]. However, with the discovery of viral carcinogenesis and the somatic mutation theory, which became the cornerstone of cancer etiology, the role of microorganisms in oncology was largely overlooked [9]. Advancements in high‐throughput sequencing and multiomics technologies have challenged the historical assumption that most internal organs are sterile. Studies of diverse malignancies, including pancreatic, lung, ovary, and breast, have demonstrated that tumor tissues often harbor distinct microbial populations compared with their corresponding normal adjacent tissues [10, 11]. This technological progress has greatly enhanced research on the link between microorganisms and cancer, uncovering the microbiota's influence on tumors. The intratumoral microbiota is now recognized as a key component of the tumor microenvironment (TME), encompassing microbial communities such as bacteria, fungi, viruses, and bacteriophages within tumor tissues. These microbes not only colonize cancer cells but are also widely present in tumor‐infiltrating immune cells [11]. This discovery significantly challenges traditional concepts, extending beyond the earlier focus on the gut microbiota's impact on gastrointestinal cancers and prompting investigations into the microbial effects in systemic malignancies [12, 13, 14]. Importantly, distinguishing correlative associations from causal mechanisms in microbiota–tumor interactions remains a central challenge. While numerous studies have demonstrated microbial associations with cancer progression, establishing causality requires stringent criteria including germ‐free animal models, and interventional studies that fulfill molecular and ecological causality frameworks [15].

Emerging evidence has demonstrated that microbiota influence host–microbiota–tumor interactions through various mechanisms, exerting both procancer and anticancer effects [15, 16, 17]. Certain microorganisms act as direct oncogenic factors, such as *Helicobacter pylori* in gastric cancer (GC) and Epstein–Barr virus (EBV) in nasopharyngeal cancer [18, 19]. Additionally, microorganisms can indirectly affect cancer progression by inducing DNA damage, activating oncogenic pathways, and triggering inflammatory responses [20, 21, 22, 23]. Microbiota also regulate chemokine secretion, antigen presentation, and immune cell infiltration, thereby promoting the formation of tertiary lymphoid structures, which are positively correlated with responses to immune checkpoint blockade (ICB) therapy [24]. As a result, microbiota‐driven cancer management has garnered increasing attention [25]. The clinical relevance of microbiota‐based interventions is increasingly supported by ongoing clinical trials. For instance, fecal microbiota transplantation (FMT) combined with anti‐PD‐1 therapy has shown promising results in clinical trials for melanoma (NCT03353402) and non‐small cell lung cancer (NSCLC) (NCT04951583), where patients who received FMT from responders demonstrated improved ICB outcomes [26, 27]. Similarly, probiotic interventions are being evaluated in combination with ICB in multiple Phase I/II trials (NCT03817125, NCT03341143) [28, 29]. These clinical investigations provide foundational evidence supporting the therapeutic potential of microbiota‐based cancer therapies, although larger randomized controlled trials are still needed to establish definitive efficacy.

The microbiome's potential as a noninvasive biomarker is gaining recognition [30]. A pan‐cancer integrative analysis of 2839 samples employing 16S rRNA sequencing has identified distinct intratumoral microbial signatures across nine cancer types, establishing microbial community profiling as a diagnostic and prognostic biomarker with cross‐cancer applicability [31]. For gastrointestinal malignancies, noninvasive approaches including tongue coating microbiota analysis, fecal fungal metagenomic signatures, and circulating microbial metabolite profiling are being systematically developed for the early detection of colorectal, pancreatic, and GCs, with microbe‐derived metabolites playing key roles in both tumorigenesis and modulation of immunotherapy responses [32, 33, 34, 35, 36]. In hormone‐related cancers, the gut microbiota influences breast cancer risk through estrogen metabolism and chronic inflammation, and compositional microbial patterns have demonstrated value as noninvasive diagnostic and prognostic biomarkers for patient stratification [37]. In prostate cancer, shifts in circulating microbial metabolites offer emerging candidate markers for liquid biopsy‐based early detection [38]. Collectively, the convergence of microbiome profiling, multiomics integration, and machine learning is advancing precision medicine, wherein individual microbial signatures are poised to guide both diagnosis and personalized therapeutic interventions across diverse cancer.

The intricate relationship between the host microbiota and cancer represents one of the most dynamic frontiers in oncology. In this review, we have systematically dissected the host–microbiota–tumor axis, spanning tissue‐specific colonization patterns, molecular mechanisms of microbial signaling, immune and metabolic modulation, and emerging therapeutic strategies. The accumulating evidence firmly establishes that the microbiota is not merely a bystander but an active participant in cancer initiation, progression, and response to therapy. However, several critical challenges must be addressed before microbiota‐based approaches can be translated into routine clinical practice.

## 2 The Cancer‐Associated Microbiota: Niches and Diversity

The human microbiome niches refer to the specific environments provided by various parts of the body for microbial colonization [39, 40]. Although these niches are physically connected, their distinct physiological conditions, nutrient availability, and immune defenses shape the composition and function of their respective microbial communities [41, 42]. Microorganisms begin colonizing the body at birth, demonstrating diversity and site‐specificity across anatomical locations such as the gut, oral cavity, skin, respiratory tract, and vagina [43, 44, 45]. Through continuous adaptation, these microorganisms establish stable populations within these spatially separated niches, which in turn regulate local physiological functions. Furthermore, interactions between distant niches, facilitated by metabolic exchanges and microorganism transfer, collectively contribute to homeostasis maintenance or the development of diseases [46, 47]. Microorganisms from one organ‐specific niche can migrate to others, encountering changes in pH, temperature, toxins, and immune cell composition [48, 49]. While cross‐niche migration can challenge the microbial communities of various organs or tissues, the overall composition of microorganisms remains relatively stable [50]. The microenvironment specific to each organ supports the stability of its associated microbiota, helping to prevent unintended disruptions [51, 52].

The tissue‐specificity of microbial colonization across different malignancies is increasingly recognized. Breast cancer tissues harbor particularly high microbial richness and diversity with distinct compositional patterns, while lung cancer tissues show enrichment of oral commensals such as Streptococcus and Veillonella [11, 53]. Pancreatic ductal adenocarcinoma (PDAC) displays a distinctive intratumoral microbiome overrepresented by Proteobacteria and Actinobacteria [10]. Ovarian cancer tissues exhibit unique microbial signatures, and colorectal cancer (CRC) is characterized by enrichment of *Fusobacterium nucleatum* (*F. nucleatum*), *Bacteroides fragilis* (*B. fragilis*), and *Escherichia coli* (*E. coli*) [20, 22, 54]. These tissue‐specific patterns underscore the importance of organ‐specific microbial ecology.

## 3 Gut Microbiota: Composition, Function, and Systemic Impact

**FIGURE 1:**
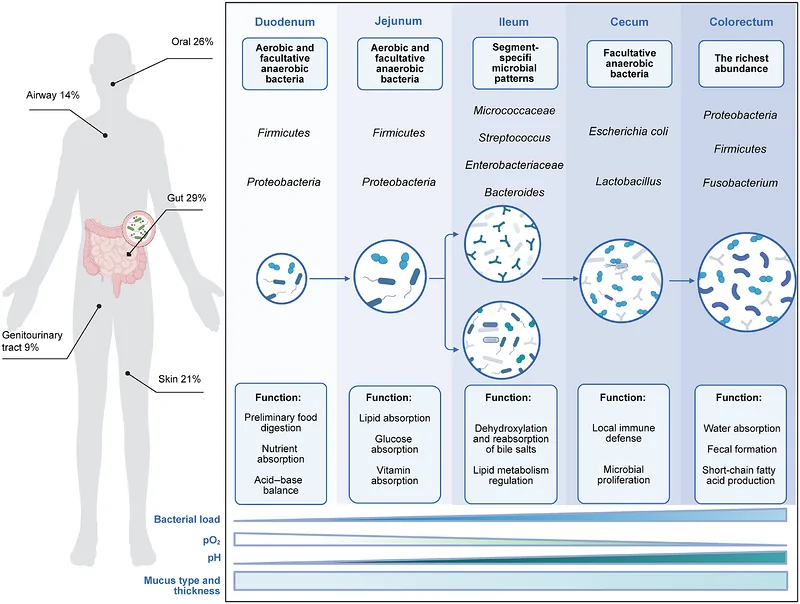
Anatomical distribution and functional organization of the human microbiota. Microbial biomass and composition vary across oral, airway, gut, genitourinary, and skin niches. Along the intestine, segment‐specific communities support nutrient absorption, bile‐acid transformation, barrier immunity, and short‐chain fatty acid production, with the greatest microbial biomass in the colorectum.

The gut harbors a highly diverse microbial community, containing approximately 4 × 10^14^ microorganisms, with the colon being the primary contributor to the total bacterial count [55, 56]. The microbial composition and function exhibit significant heterogeneity along the anatomical length of the gut [57, 58]. Yang et al. summarized the distributional and functional characteristics of gut microbiota across different intestinal segments: the duodenum is primarily dominated by aerobic and facultative anaerobic bacteria, such as *Firmicutes* and *Proteobacteria*, which contribute to initial food digestion, nutrient absorption, and acid–base balance [59]; the jejunum, exhibiting microbial diversity between the duodenum and ileum, aids in lipid, glucose, and vitamin absorption; the ileum displays segment‐specific microbial patterns, with *Micrococcaceae* and *Streptococcus* abundant in the proximal regions, while *Enterobacteriaceae* and *Bacteroides* dominate the distal segments, contributing to bile salt dehydroxylation, reabsorption, and regulation of lipid metabolism; the cecum is mainly populated by facultative anaerobes like *Escherichia coli* and *Lactobacillus*, supporting local immune defense and microbial proliferation; finally, the colorectal microbial community, characterized by *Firmicutes*, *Bacteroidetes*, *Proteobacteria*, *Verrucomicrobia*, *Fusobacterium*, and *Actinomycetes*, plays a central role in water absorption, fecal formation, and short‐chain fatty acid (SCFA) production (Figure 1).

Functioning as a “one organ,” the gut microbiome regulates the physiological and pathological processes of multiple organs through the gut‐organ axis [60]. By interacting with the central nervous system via the gut–brain axis, the gut microbiome influences the onset and progression of neurodegenerative and neuropsychiatric diseases, mediated by neuroinflammation, microbial metabolites, and vagus nerve signaling [61, 62]. Within the immune system, the gut microbiome affects both innate and adaptive immune responses, maintaining immune tolerance and influencing autoimmune diseases as well as tumor immune surveillance [63, 64]. Dysbiosis in gut microbiome–immune interactions can promote chronic inflammatory states [65]. Additionally, the gut microbiome plays a crucial role in metabolic diseases, such as obesity, Type 2 diabetes, and nonalcoholic fatty liver disease, by regulating host energy balance, insulin sensitivity, and lipid metabolism [66]. It also impacts the circulatory system, linking the gut microbiome to cardiovascular conditions like atherosclerosis and hypertension [67]. The gut–lung axis further demonstrates the influence of the gut microbiome on the respiratory system, with early dysbiosis associated with an increased risk of asthma and chronic obstructive pulmonary disease [68]. In the digestive system, an imbalanced gut microbiome can directly impair the mucosal barrier and trigger abnormal immune activation, contributing to conditions such as inflammatory bowel disease and CRC [60]. Moreover, the gut–kidney axis accelerates the progression of chronic kidney disease through the accumulation of uremic toxins and systemic inflammation [69]. The gut–reproductive axis has been implicated in conditions like polycystic ovary syndrome and pregnancy complications [70]. The gut–skin axis is also involved in the pathogenesis of inflammatory skin conditions such as atopic dermatitis and psoriasis [71]. Recent studies have expanded our understanding of the gut microbiome's role in musculoskeletal diseases, with the gut–joint and gut–muscle axes influencing conditions like rheumatoid arthritis, osteoporosis, and sarcopenia [72, 73].

## 4 Intratumoral Microbiota: Existence, Colonization, and Distribution

Recent advances in TME research have highlighted the microbiome as a potential component of tumor tissues. For years, tumor tissues were believed to be sterile; however, recent advancements in molecular detection and imaging technologies have challenged this assumption [74]. Nejman et al. conducted a large‐scale microbiome analysis across seven cancer types, providing the first evidence of bacterial colonization within cancer cells and tumor‐infiltrating immune cells [11]. Fu and colleagues further demonstrated that bacteria colonizing breast cancer cells promoted tumor metastasis [75]. Additionally, fungal histological staining across multiple cohorts confirmed the presence of fungi in tissue microarrays from various cancers, underscoring the complexity and diversity of the intratumoral microbiome [76].

However, it must be noted that the study of the intratumoral microbiome is particularly challenging due to low‐biomass contamination artifacts. Emerging spatial multiomics technologies have significantly advanced the rigorous validation of intratumoral microbiota. Spatial transcriptomic enables the simultaneous mapping of host gene expression and microbial RNA within intact tissue sections, providing direct evidence of microbial localization at the transcriptional level [77]. Multiplex immunofluorescence combined with specific antibacterial antibodies or rRNA‐targeted probes allows visualization of microbial cells and their spatial relationship with immune cells and tumor cells at subcellular resolution [78, 79, 80]. Recent application of GeoMx digital spatial profiling has revealed that intratumoral bacteria are frequently localized within immune cell‐rich regions rather than randomly distributed, suggesting active immune–microbe interactions within the TME [81]. These spatial approaches, combined with laser capture microdissection coupled with 16S rRNA sequencing, are beginning to bridge the gap between bulk sequencing correlations and the actual three‐dimensional architecture of microbial colonization in tumors. However, whether these signals in immune cells represent live intracellular bacteria capable of replication, phagocytosed bacterial debris, or translocated microbial nucleic acids remains an active area of investigation. Rigorous validation with culture‐based methods and functional studies is necessary to distinguish viable intracellular bacteria from passive uptake of microbial products. Therefore, caution is warranted when discussing the presence of bacteria within immune cells.

Tumor tissues attract microbial colonization, including bacteria, fungi, and other microorganisms, primarily through three mechanisms: (I) abnormal angiogenesis, characterized by loose connections between vascular endothelial cells and increased permeability, facilitates microbial extravasation and colonization in tumors [82]; (II) an immunosuppressive TME weakens pathogen clearance, creating a favorable niche for microbial survival and proliferation [83]; and (III) the hypoxic and acidic conditions of tumors promote the colonization and growth of anaerobic or facultative anaerobic microorganisms, providing optimal conditions for microbial expansion [84].

Altogether, the particularity of tumor tissues is conducive to the colonization of microbes. And the intratumoral microbiota exhibits remarkable spatial and compositional heterogeneity within the TME, with distinct microbial communities colonizing different regions of the same tumor. This heterogeneity might be related to regional variations in oxygenation, pH, nutrient availability, and immune cell infiltration. Understanding the existence and spatial heterogeneity of microbes is crucial for developing effective therapeutic strategies, as regionally distinct microbial populations may differentially influence local immune responses and treatment sensitivity.

## 5 Microbial Signatures Across Tumor Types: Low Biomass and Compositional Heterogeneity

Intratumoral microbiomes have become a prominent focus in oncology and microbiology, primarily due to their low biomass. Although the presence of microbiomes in tumors was recognized over a century ago, their low abundance in tumor tissues and susceptibility to exogenous contamination were only highlighted through high‐throughput sequencing technologies [85]. Despite their low biomass, these microbiomes influence the TME through pathogen‐associated molecular patterns (PAMPs) and specific metabolites [86]. A critical question in the field concerns the abundance threshold at which low‐biomass microbiota become functionally relevant. Current research does not clearly explain this issue. However, picomolar concentrations of lipopolysaccharide (LPS) are sufficient to activate TLR4 signaling, suggesting that the functional impact of intratumoral bacteria may be mediated through potent immunostimulatory molecules rather than requiring high bacterial loads [87, 88]. Therefore, it is inferred that the functional threshold of intratumoral microbiota is not defined by absolute bacterial counts. The local concentration of bacterial effector molecules reaching active levels may be more important for their function.

Consequently, enhancing detection techniques for low‐biomass microbiomes has become crucial for advancing research in this area. The composition of intratumoral microbiomes exhibits significant heterogeneity and diversity across different cancer types. A comprehensive study by Nejman et al. across seven cancer types revealed notable differences in microbial community structures among various cancers [11]. Notably, breast cancer tissue demonstrated particularly high microbial richness and diversity compared with other cancers. At the phylum level, *Proteobacteria* and *Firmicutes* were predominant in CRC, while *Actinobacteria* consistently showed higher relative abundance in nongastrointestinal cancers such as lung, breast, and ovarian cancers. This phylum‐level divergence reflects distinct microenvironmental selection pressures and functional contributions to carcinogenesis. Most of *Proteobacteria*, particularly *Enterobacteriaceae*, are facultative anaerobes capable of thriving under both oxygenated and hypoxic conditions [89]. The inflammatory and hypoxic TME of solid tumors provides them a competitive advantage over other microbes. In contrast, the dominance of *Actinobacteria* in breast cancer likely stems from the mammary gland's anatomical derivation from skin adnexal structures. *Actinobacteria* are core members of the skin microbiome and can access breast tissue via ductal system [90, 91]. Thus, the intratumoral microbiome composition across cancer types appears to be shaped by a combination of tissue‐specific anatomical niches and local microenvironmental selection pressures.

## 6 Origins and Routes of Microbial Entry Into Tumors

There are several routes through which tumor‐associated microbiota colonize tumor tissues, including damaged epithelial barrier, dissemination of adjacent normal tissues, and distal transmission of blood and lymphatic circulation. Several studies have shown that the microbial composition in tumor tissue is highly similar to that of its paired normal neighbors, suggesting that local microbial spread may be present [92]. However, some studies have suggested that microorganisms may migrate from tumors to surrounding normal tissues, and this hypothesis still needs to be further verified by spatial multiomics techniques [93]. The colonization of distal cancers, such as liver or breast cancers that not directly connected to the external environment, implies that bacteria must traverse host barriers and travel through the body. The oral cavity is one of the main routes for systemic dissemination, especially through the oral–digestive axis. *F. nucleatum* is ubiquitous in the oral cavity but is a major pathogen in CRC. It reaches the colon through two completely different routes: one is the intestinal route, that is, it reaches the intestine by swallowing and tolerating stomach acid; the second is the hematogenous route, that is, periodontal pathogens enter the bloodstream during transient bacteremia [94, 95]. Once in circulation, its surface adhesin FadA binds to vascular E‑cadherin, disrupting tight junctions to enhance endothelial permeability and promote bacterial transmigration into well‑vascularized tissues such as CRC [96]. Similarly, the oral–pancreatic axis facilitates the translocation of oral bacteria such as *P. gingivali* to the pancreas via the bloodstream or through biliary/duodenal reflux [97, 98]. In the respiratory tract, micro‐aspiration, recognized as the main route of the nasal–pulmonary axis, mediates the entry of nasopharyngeal and oral microbiota into the lungs [99, 100]. Disruption of the gut–vascular barrier allows intestinal bacteria to enter the portal vein, a key mechanism for liver metastases [101]. A key question is how do these bacteria survive immune clearance during cyclic transport. The “Trojan horse” phenomenon wherein bacteria actively hijack immune cells as armored transport carriers potentially provides the answer [102]. *F. nucleatum* can survive in human neutrophils by inhibiting oxidative bursts, effectively utilizing these cells to protect *F. nucleatum* from immune killing and thus migrate into the tumor tissues [103]. Chen et al. further revealed that *F. nucleatum* reduced intracellular reactive oxygen species (ROS) and generates H_2_S to survive, thereby enabling its persistence within neutrophils and promoting CRC metastasis [104]. Macrophages are also utilized. Intracellular pathogens like *Listeria monocytogenes* have evolved sophisticated mechanisms to ensure their survival in macrophages for transport [105]. Furthermore, *F. nucleatum* has been shown to accumulate within M2‐type macrophages in human CRC samples, suggesting the possibility that these bacteria may be implicated in the induction of M2‐like polarization [106]. Despite these mechanistic insights, the fraction of circulating *F. nucleatum* that utilizes this intracellular route remains poorly characterized. Quantitative estimates of the prevalence of neutrophil‐ or macrophage‐associated *F. nucleatum* in the bloodstream relative to extracellular bacteria are currently lacking. Determining this proportion will be essential for understanding the relative contribution of phagocyte‐mediated dissemination to *F. nucleatum* tumor colonization and for designing strategies to intercept this pathway. Additionally, Fu et al. demonstrated that intracellular microbiota within breast cancer promotes metastatic colonization by enhancing tumor cell survival to mechanical fluid shear stress [75].

Emerging evidence indicates that microbial communities both at primary tumor sites and within distant organs play a critical role in pre‐establishing the premetastatic niche (PMN) to facilitate metastatic colonization [107, 108]. Specific microorganisms reshape the microenvironment of distal organs before cancer cells arrive. For example, *F. nucleatum* isolated from liver metastases are identical to those in the primary CRC of the same patient, indicating that the bacteria in the cancer cells facilitates the protumorigenic microenvironment in the new organ [109]. Gut bacteria translocate to the liver through the gut–liver axis and initiate a proinflammatory response in the liver that enhances PMN formation [101, 110]. The PMN concept is well‐illustrated across several cancer types. Within the gut–liver axis, dysbiosis‐induced disruption of intestinal barrier integrity enables microbial translocation that triggers a cascade of hepatic changes including inflammation, immune suppression, metabolic reprogramming, and vascular remodeling [111, 112]. As a key bacterium for immune‐inflammatory remodeling in liver metastasis of CRC, *F. nucleatum* adheres to metastatic lesions via the Fap2–Gal–GalNAc interaction [113, 114]. It downregulates CD8^+^ T cells and FOXP3^+^ regulatory T (Treg) cells to establish an immunosuppressive microenvironment and binds TIGIT receptor on NK cell through Fap2 to inhibit NK cytotoxicity and mediate innate immune evasion, thereby facilitating liver metastasis [115, 116]. Beyond *F. nucleatum*, a pan‐cancer comparative analysis of tumor microbiota across colorectal, lung, and breast cancers has identified a metastasis‐specific bacterial signature with *Streptococcus spp*. as the predominant microorganisms [117]. Similarly, microbial dysbiosis and its derived bioactive substances trigger pulmonary inflammatory responses, activate multiple pattern recognition receptor (PRR) pathways, and recruit immunosuppressive immune cells, which drive the establishment of lung PMN [118]. Additionally, enterotoxigenic *Bacteroides fragilis* (ETBF) in breast cancer triggers the secretion of proinflammatory cytokines and chemokines, which indirectly attract immunosuppressive cells to PMN and reshape the local microenvironment to create a permissive milieu for tumor metastatic spread [119]. Meanwhile, chronic pulmonary infections, particularly those caused by *Pseudomonas aeruginosa*, have been found to enhance breast cancer lung metastasis by recruiting MHC‐II^hi^ neutrophils into the lungs prior to metastatic colonization [120]. These diverse colonization pathways suggest that targeting microbial migration could offer a novel strategy for influencing tumor progression.

## 7 Methods for Microbiome Detection and Characterization in Cancer

In microbial detection, “omics” refers to a range of high‐throughput analytical techniques used to comprehensively characterize microbial composition and function, including genomics, transcriptomics, metabolomics, proteomics, and multiomics approaches [121, 122]. These techniques enable large‐scale data collection and analysis, offering a holistic view of microbial community structure, metabolic pathways, and host interactions in a culture‐independent manner [123, 124]. This overcomes the limitations and biases inherent in traditional culture‐based methods. The 16S ribosomal RNA (16S rRNA), universally present in both bacteria and archaea, is widely used as a key marker for identifying prokaryotic microbial diversity [125, 126, 127]. The 16S rRNA contains both highly conserved regions, facilitating the design of universal primers, and hypervariable regions, which allow differentiation between species [128, 129]. By amplifying and sequencing these hypervariable regions using high‐throughput platforms, 16S rRNA sequencing enables phylogenetic analysis and taxonomic classification [128, 130]. However, 16S rRNA sequencing alone is often insufficient to accurately characterize the metabolic potential or activity of microbial communities, as it targets housekeeping genes rather than functional ones [131, 132]. Metagenomics, which analyzes the entire genome, requires deeper sequencing and tends to be more expensive [133]. Unlike 16S rRNA sequencing, metagenomics provides accurate species or even strain‐level identification and uncovers previously unknown or uncharacterized functional potentials [134, 135]. Metatranscriptomics, on the other hand, addresses microbial transcriptional activity, revealing differences that cannot be detected through metagenomic studies alone [136, 137]. The primary challenge with metatranscriptomics is that rRNA constitutes the majority of total RNA, necessitating selective removal of rRNA to enhance the relative abundance of mRNA and enable precise quantification of functional transcripts. He et al. compared two rRNA removal methods, finding that subtractive hybridization introduced the least bias, whereas nuclease digestion may compromise the accuracy of mRNA abundance [138]. Metaproteomics, which utilizes high‐resolution mass spectrometry, analyzes the expressed proteome of microbial communities, shedding light on their functional characteristics and identifying proteins with diagnostic or mechanistic significance [139, 140]. Additionally, metabolomics examines small‐molecule metabolites, offering direct insights into microbial metabolic activity [141, 142]. When integrated, these approaches provide functional insights that DNA‐ and RNA‐centric methods alone cannot offer [143].

Fluorescence in situ hybridization (FISH) employs fluorescence‐labeled nucleic acid probes that specifically bind to bacterial 16S rRNA or conserved fungal 28S rRNA sequences, enabling in situ detection, identification, and quantification of microbial pathogens [144, 145, 146]. By providing direct evidence at the cellular level, FISH overcomes the limitations of other techniques regarding spatial localization and intracellular distribution. When combined with other detection technologies, such as 16S rRNA sequencing, FISH allows for more comprehensive analysis. Immunohistochemistry (IHC) targets microbial biomarkers, including surface proteins and secretions, aiding in the validation of microbial presence and distribution [147, 148]. However, interference from fluorescent signals restricts FISH and IHC's ability to detect multiple microorganisms simultaneously. Traditional microscopic imaging is hindered by lengthy detection cycles and limited accuracy, which constrains its ability to examine large samples or rare microbial species. In contrast, the integration of computer image analysis has enabled efficient, fully automated, and precise detection [149]. Transmission electron microscopy (TEM), with its nanometer‐scale resolution, is particularly useful for viral detection and bacterial identification research [150, 151, 152, 153]. Correlative light and electron microscopy (CLEM) combines the targeted labeling and spatial localization capabilities of light microscopy with the high resolution of electron microscopy, allowing for precise visualization and analysis of target structures in biological samples [154]. The use of advanced probes makes CLEM especially suitable for tracking microbial distribution, intracellular behavior, and enhancing imaging precision of disease‐associated microorganisms [155, 156].

**FIGURE 2:**
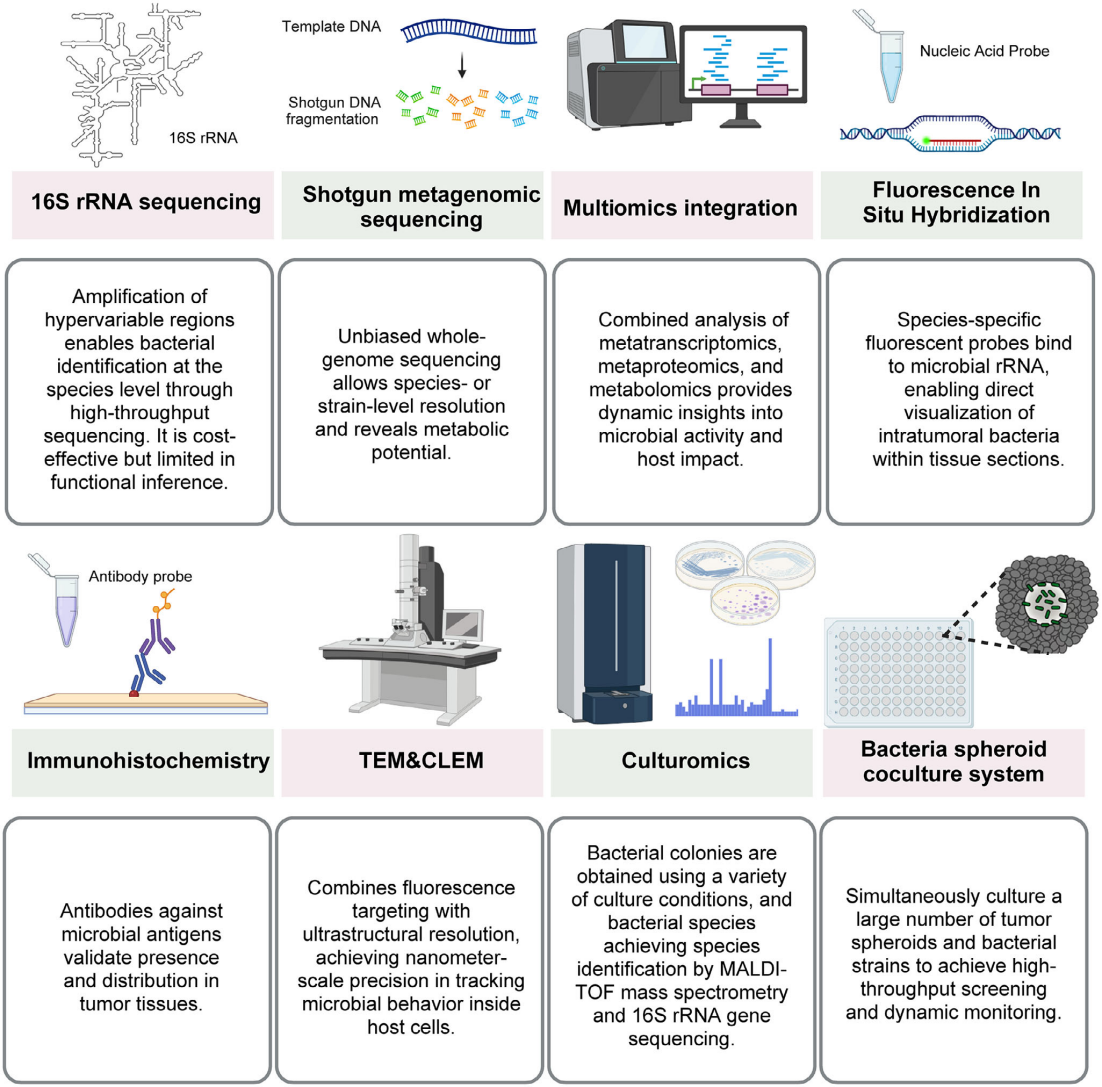
Methods for microbiome detection and characterization in cancer. Sequencing and multiomics approaches define community composition and function; FISH, immunohistochemistry, TEM, and CLEM provide spatial validation; and culturomics and bacteria‐spheroid coculture enable functional testing of viable organisms.

Traditional microbial culture methods often face challenges in effectively isolating live bacteria from tissues due to limitations such as low biomass and strict growth requirements [157]. However, the advancement in culturomics has enabled the successful isolation of cultivable commensal bacteria from various solid tumors, including melanoma and nasopharyngeal carcinoma [158, 159]. An innovative in vitro model, the bacteria spheroid coculture (BSCC) system, has been develop to simulate the complex interaction between microorganisms and cancer cells in the three‐dimensional space [160]. This system involves coculturing three‐dimensional tumor spheroids with specific bacterial strains in low‐adhesion plates, which maintain spheroid integrity and enable bacteria to selectively invade and colonize the spheroids. The BSCC model allows for the investigation of bacteria impacts on the tumor behavior, as well as the evaluation of bacterial interactions with chemotherapy or immunotherapy agents. Furthermore, the integration of automated high‐throughput bacterial isolation platforms with machine learning strategies has significantly improved the efficiency and reproducibility of isolating live bacteria from tumor tissues [161]. However, it is important to acknowledge a key limitation of the BSCC system and culturomics. Specifically, these approaches are inherently biased toward cultivable organisms. Current culture conditions cannot replicate the complex nutritional and ecological requirements of many commensal microorganisms, probably overlooking functionally important but unculturable taxa. Therefore, culturomics‐based approaches should be viewed as complementary to culture‐independent methods (Figure 2).

## 8 Functional Framework of Host–Microbiota–Tumor Interactions

The role of the microbiota in cancer biology has surpassed the traditional notion that diseases are caused by single pathogens and has now acknowledged as a complex regulatory network. Before delving into specific signaling pathways and metabolic mechanisms, it is essential to firstly elucidate the functional characteristics of the microbiota as initiators, regulators, and therapeutic effectors in the biological processes of cancer development. It is indicated that microorganisms not only undermine the stability of the host genome through direct genotoxic effects but also promote the occurrence and progression of cancer through various mechanisms, inducing chronic inflammation, reshaping the epigenetic landscape, and facilitating molecular mimicry.

### 8.1 Microbes as Tumor Initiators

Tumor initiators are microorganisms that directly cause normal cells to become cancerous. As initiators, microorganisms induce cancer mainly by triggering genotoxicity, epigenetic reprogramming, and causing chronic inflammation.

#### 8.1.1 Direct Genotoxicity and DNA Damage

Genomic instability is one of the most significant markers of cancer [162]. Specific microorganisms have been shown to directly induce host DNA damage, thereby acting as endogenous mutagens. Genotoxicity is mainly achieved through specific toxins secreted by bacteria, which can directly attack the double helix structure of DNA, leading to single‐strand or double‐strand breaks (DSBs) [163]. *Escherichia coli* carrying the *pks* genomic island (*pks*+ *E. coli*) is capable of producing colibactin [164]. This toxin can form DNA interstrand cross‐links and damages the DNA double helix, leading to genome instability [165]. If such damage is not properly repaired, it can lead to specific genetic mutations, such as base substitutions (SBS88) and insertion/deletion mutations (ID18). These distinct mutational patterns serve as a molecular “fingerprint” of exposure to the colibactin. By injecting *pks*+ *E. coli* into human intestinal organoids, Clevers et al. detected this particular fingerprint in the CRC genomes, thereby confirming that colibactin can directly induce carcinogenic mutations [166]. Crucially, the failure to adequately resolve this DNA damage leads to profound genomic aberrations and chromosomal instability. This specific failure acts as the primary starting point for colibactin‐driven cancer [167]. It has been reported that *pks*+ *E. coli* concurrently downregulated the ataxia–telangiectasia mutated checkpoint pathway, which prevented normal cell cycle arrest and allows mutated hepatocytes to survive and multiply [168, 169]. Cytolethal distending toxin (CDT), a toxin secreted by various Gram‐negative bacteria, is a heterotrimeric toxin [170]. Its active subunit CdtB possesses activity similar to that of DNase I, which directly cuts DNA and result in the formation of DSBs [171]. Although cells attempt to repair this damage, repair mechanisms may fail during chronic infection. This leads to the failure of cellular checkpoints, allowing cells to continue dividing with carrying unrepaired mutations, ultimately facilitating malignant transformation [172]. In an aseptic mouse model, infection with the human clinical isolate *Campylobacter jejuni* 81–176 was found to possess a significant promoting effect on the development of CRC [173]. Additionally, *Bacteroides fragilis* toxin (BFT) secreted by ETBF not only disrupts the epithelial barrier but also induces the high expression of spermine oxidase (SMOX) [174, 175]. During the decomposition of polyamines, SMOX generates by‐products such as hydrogen peroxide and ROS [176]. These high concentrations of ROS directly attack DNA, causing oxidative damage and cumulative mutations, and serve as a powerful initiating signal for the carcinogenesis of colonic epithelial cells.

#### 8.1.2 Reshaping Epigenetics

In addition to directly altering DNA sequences, microorganisms also profoundly influence the expression patterns of host genes through epigenetic mechanisms. This regulation does not involve changes in DNA sequences but is achieved through DNA methylation, histone modifications, and the regulation of noncoding RNAs [177]. Microbial metabolites serve as a crucial bridge connecting the microbiota with the host's epigenetic modifications. For instance, SCFAs produced by gut microbiota, especially butyric acid, are potent histone deacetylase (HDAC) inhibitors [178]. Under normal physiological conditions, butyric acid helps maintain the homeostasis of epithelial cells [179]. It was also found that the colonization of specific microorganisms is closely related to the methylation state of the host genome. The high abundance of *F. nucleatum* is positively correlated with the CpG island methylation phenotype, which is often accompanied by high methylation and transcriptional silencing in the promoter region of key tumor suppressor genes such as MLH11 [180, 181]. However, it is imperative to highlight that current evidence establishes a robust correlation between *F. nucleatum* and CpG island methylation rather than explicit causality. Although the potential capacity of *F. nucleatum* to directly drive DNA methylation changes requires further exploration, *F. nucleatum* appears to closely interact with the posttranscriptional epigenetic modification through noncoding RNA networks [182]. For instance, it can upregulate oncogenic miR‐21 to enhance CRC cell proliferation and tumor growth [183]. Concurrently, it induces the expression of miR‐31, which targets the eIF4EBP1 and eIF4EBP2. This suppression of translational inhibitors disrupts downstream protein synthesis and cell proliferation, potentially contributing to intestinal carcinogenesis [184]. In addition, pathogenic infection can also change the activity of m6A RNA methylation modifier in host cells, affecting the stability and translation efficiency of transcripts [185].

#### 8.1.3 Chronic Inflammation

Microorganisms and their products, such as LPS and flagellin, act as PAMPs, continuously activating PRRs on the surface of host cells [186]. This continuous stimulation leads to the activation of inflammatory core transcription factors such as nuclear factor κB (NF‐κB) and STAT3, thereby inducing the sustained release of proinflammatory factors. Unlike acute inflammation aimed at eliminating pathogens, this microbe‐driven chronic inflammation not only fails to inhibits cancers but promotes cancer progression by reshaping TME [187].

Microbe‐driven chronic inflammation varies substantially across tissues and species. These tissue‐specific microbial profiles arise from differences in anatomical location, oxygen tension, nutrient availability, and immune surveillance architecture. Consequently, the inflammatory response to the same bacterial stimulus can differ markedly across tissues. For instance, the oral mucosal immune system exhibits a spatially distinct organization compared with the gut [188]. The mechanisms by which gut‐derived microbial metabolites orchestrate immunity in distant organs, such as lung, liver, and brain, are fundamentally different from local inflammatory responses at the intestinal mucosa [189]. In the context of *F. nucleatum*, the inflammatory consequences of this bacterium within the hypoxic, metabolically distinct colorectal TME are unlikely to be identical to those in the oral cavity or other anatomical sites. The clinical translation is also constrained by species‐specific differences. Mouse models, while invaluable for mechanistic studies, possess gut microbiomes that differ substantially from humans in phylum‐level composition [190, 191]. Furthermore, their immune systems are far more tolerant to LPS than human immune systems (sensitivity differences can exceed 100,000‐fold), owing in part to species‐specific variations in TLR4/MD‐2 recognition and downstream signaling pathways [192]. As a result, microbe‐driven inflammatory signaling cascades identified in murine models may not fully recapitulate the human condition. These tissue‐ and species‐specific differences underscore the importance of placing microbe‐driven inflammation within the specific anatomical and immunological environments rather than assuming universal mechanisms.

### 8.2 Microbes as Tumor Regulators

During the development of cancer, microorganisms may no longer be the direct cause of carcinogenesis, but influence the growth and invasiveness of cancers by regulating the immune responses, metabolic characteristics, and matrix properties of TME. Regulators can either be located in the intestine to exert their effects through distal signals or colonize within the tumor to exert direct regulatory function.

#### 8.2.1 Regulating Immune Response

The intestine is an important immune organ in the human body and is inhabited by the vast majority of microorganisms. The gut microbiota does not directly contact cancers located in distal organs, but regulates the immune responses throughout releasing metabolites and signaling molecules that can enter the blood circulation. Inosine produced by *Bifidobacterium pseudolongum* can bind to the adenosine A2A receptor on the surface of T cells. In the presence of costimulatory signals provided by immune checkpoint therapy, inosine can significantly enhance the differentiation and antitumor functions of Th1 cells [193]. However, these preliminary observations demand rigorous appraisal. Current experimental evidence is limited, predominantly confined to specific murine models [194]. While these initial in vivo findings effectively outline a potential immunomodulatory pathway, the translational efficacy of *B. pseudolongum* and inosine in regulating tumor immunity within highly heterogeneous clinical oncological settings requires extensive prospective validation. The most novel finding is that cancer cells can present the peptides of intracellular bacteria on the cell surface. Through the analysis of 16S rRNA gene sequencing and HLA peptidomics, Kalaora and colleagues confirmed that melanoma cells can present peptides from intracellular bacteria to human leukocyte antigen (HLA)‐I and HLA‐II molecules [195].

#### 8.2.2 Reprogramming Metabolism

Metabolic reprogramming in tumors refers to the process by which tumor cells alter their metabolic patterns to adapt to the microenvironment, thereby meeting the bioenergetic and biosynthetic demands of rapid proliferation and survival. This reprogramming involves dysregulation across multiple metabolic pathways, collectively promoting tumor growth, invasion, angiogenesis, and therapeutic resistance [196, 197]. Oral squamous cell carcinoma (OSCC) cells exhibit intrinsically high glycolytic activity driven by constitutively elevated GLUT1 expression. *F. nucleatum* amplifies this pre‐existing glycolytic program by promoting GLUT1 plasma membrane accumulation through the GalNAc–autophagy–TBC1D5 signaling axis, further enhancing glucose uptake and lactate deposition in the TME [198]. Several viruses such as MCPyV, HCMV, and KSHV regulate aerobic glycolysis and fatty acid synthesis [199, 200, 201, 202].

#### 8.2.3 Molecular Mimicry

Molecular mimicry is an emerging and key mechanism by which the microbiota affects tumor immune surveillance. It is evident that the human microbiota encodes a substantial number of genes, which far exceeds the number encoded by the human genome. Consequently, there is a high degree of sequence homology and conformational similarity between microbial antigens and tumor‐associated antigens or auto‐antigens. Sequence homology refers to the high similarity in amino acid sequences between microbial antigens and host antigens [203]. At the conformational level, microbial antigens often share highly similar three‐dimensional structures with host antigens, allowing them to bind to the same HLA molecules and thereby be recognized by T cells [204]. Systematic analyses of the human T‐cell epitope repertoire against microbial proteomes have revealed that counterparts for over 90% of human HLA‐I ligands and 5% of HLA‐II ligands exist within commensal and pathogenic microbiota. Ragone et al. independently conducted a systematic screen for sequence homology between published tumor‐associated antigens and microbiota‐derived epitopes, revealing extensive homology including three cases of 100% linear sequence identity [203]. Dedicated resources such as the MicroEpitope database now catalogue immune epitopes derived from cancer microbiomes across multiple tumor types, which provides a systematic platform for identifying microbial peptides with potential cross‐reactivity to tumor antigens [205]. Experimental validation of these computational predictions has been achieved. Hundreds of bacteria‐derived HLA‐bound peptides have been identified in melanoma, originating from approximately 40 distinct bacterial genera and accounting for up to ∼10% of the total MHC‐I peptidome [195]. Molecular mimicry has the potential to trigger immune cross‐reactivity, which can result in two distinctive consequences [206]. A peptide derived from an *Enterococcus hirae* bacteriophage cross‐reacts with a tumor MHC class I‐restricted antigen and enhances anti‐PD‐1 efficacy in vivo. Additionally, Bessell et al. found that the epitope SVYRYYGL (SVY) expressed in the commensal gut bacterium *Bifidobacterium breve* shares sequence homology with the neoantigen SIYRYYGL (SIY) in the B16.SIY murine melanoma model [207]. They further demonstrated that T cells targeting the SVY epitope can cross‐react with this model neoantigen SIY. Through the molecular mimicry with this tumor neoantigen, the SVY epitope specifically expands and shapes a highly functional cross‐reactive CD8^+^ T cell population, thereby enhancing the host's antitumor immune surveillance even under physiological conditions. All in all, these findings establish that molecular mimicry between the microbiome and tumor antigens is structurally plausible, experimentally detectable, and functionally relevant. The breadth of bacterial genera contributing to the tumor immunopeptidome suggests that the potential for mimicry extends broadly. However, precise estimate of the proportion of bacteria engaged in functional mimicry is not yet available.

### 8.3 Microbes as Therapeutic Effectors

The term “therapeutic effectors” underscores the critical role of microbes in cancer therapy, encompassing their functions in modulating treatment responsiveness, influencing efficacy, and mediating adverse effects. This conceptual expansion elevates the microbiome from a mere pathological factor to a significant entity that can serve as both a therapeutic biomarker and a potential target for combination therapy. In chemotherapy, microbes can directly mediate drug resistance [208]. In immunotherapy, specific microbes may enhance the efficacy of PD‐1/CTLA‐4 antibodies, yet microbes are also closely linked to immunotherapy resistance [209, 210]. Beyond influencing treatment efficacy, microbes further modulate therapy‐induced toxicity [211]. Furthermore, the application of broad‐spectrum antibiotic can eliminate pathogenic bacteria. The removal of microbes often disrupts intestinal microbiota homeostasis, which may weaken the effectiveness of immunotherapy and increase the risk of immune‐related adverse events [212, 213]. Emerging intervention strategies, including engineered bacteria, oncolytic viruses (OVs), and phage therapies, aim to precisely modulate the intratumoral microecology.

## 9 Host–Microbiome–Tumor Axis: Immune and Metabolic Signaling

Microorganisms influence tumor biology through a variety of host signaling pathways. Microbial components, metabolites, and secreted factors can be sensed by immune and nonimmune cells, leading to changes in inflammatory responses, cellular metabolism, and tumor behavior. It is necessary to highlight that the consequences of microbial signaling are highly context dependent. Specifically, the same microbial stimulus may promote tumor‐associated inflammation in one setting while enhancing immune activation in another, probably depending on the responding cell type, tumor stage, and surrounding microenvironment.

### 9.1 Signaling Pathways

The signaling pathways downstream of microbial recognition do not function as isolated linear cascades but rather form an interconnected network characterized by extensive crosstalk, functional redundancy, and context‐dependent divergence. TLR (Toll‐like receptors) engagement activates NF‐κB through both myeloid differentiation factor‐88 (MyD88)‐ and TRIF‐dependent pathways, positioning NF‐κB as a central convergence hub that also integrates signals from tumor necrosis factor (TNF) receptors, interleukin‐1 (IL‐1) receptors, and genotoxic stress [214]. Usually triggered by cytosolic DNA, the cGAS–STING pathway activates NF‐κB as a secondary branch alongside its primary TBK1–interferon regulatory factor (IRF)3 axis, which creates functional overlap with TLR signaling at the level of NF‐κB‐dependent inflammation [215]. It still remains distinct in its additional induction of Type I interferon (IFN‐I) responses. Wnt/β‐catenin signaling intersects with NF‐κB via different mechanisms. β‐Catenin can physically associate with NF‐κB subunits to repress transcriptional activity, while GSK‐3β differentially modulates the two pathways, positioning β‐catenin as a molecular switch between proliferative and inflammatory gene programs [216, 217]. PI3K–AKT signaling converges with β‐catenin through AKT‐mediated inactivation of GSK‐3β, which stabilizes β‐catenin and promotes its nuclear translocation [216]. Moreover, AKT also provides an alternative route to NF‐κB activation through direct IKK phosphorylation, bypassing canonical TLR‐dependent signaling. The ERK and JAK–STAT pathways, though activated by distinct extracellular ligands, converge on shared transcriptional targets including AP‐1 and STAT‐responsive genes [218]. And ERK can directly phosphorylate STAT family members, creating additional cross‐talk. Notably, STAT3 and NF‐κB further cooperate at target gene promoters to induce inflammatory cytokines. NF‐κB‐dependent IL‐6 production feeds back to activate STAT3 and establishing a feed‐forward amplification loop that sustains chronic inflammation [219]. Together, these interactions create a signaling landscape in which the net cellular response to microbial stimulation probably depends on the combinatorial activation state of multiple pathways, the tissue context, and the duration of exposure.

#### 9.1.1 TLR Signaling

TLRs, members of the PRR family, primarily recognize PAMPs to initiate host defense mechanisms against pathogens [214]. The activation of TLR signaling triggers downstream signaling molecules through both MyD88‐dependent and MyD88‐independent pathways [220]. Specifically, the MyD88‐dependent pathway activates the IKK complex via the adaptor protein MyD88, leading to the activation of NF‐κB and MAPK, which induce the expression of inflammatory cytokines. In contrast, the MyD88‐independent pathway, also known as the TRIF‐dependent pathway, primarily activates IRF3 through TLR3/4, promoting IFN‐I production while also synergistically activating NF‐κB to enhance antiviral immunity. In various cancers, specific microbial components can influence immune responses, inflammatory processes, and tumor cell behavior by activating TLR signaling [221].

In HCC, penicillin‐binding protein 1B (PBP1B) from *Klebsiella pneumoniae* binds to TLR4 on tumor cells, activating downstream signaling pathways that promote tumor cell proliferation [222]. Similarly, in CRC, *F. nucleatum* exerts multiple protumor effects via the TLR4 signaling axis. Yang et al. reported that activation of the TLR4–MYD88 pathway by *F. nucleatum* upregulated miR‐21 expression, which suppressed RAS‐GTPase‐activating protein 1 (RAS‐GAP1) levels, thereby promoting tumor cell proliferation [183]. Chen demonstrated that *F. nucleatum* enhanced CRC growth through the IL‐6/p‐STAT3/c‐MYC cascade mediated by TLR4, which promoted M2‐like polarization of tumor‐associated macrophages [223]. Furthermore, *F. nucleatum* upregulated cytochrome P450 2J2 (CYP2J2) and its metabolite 12,13‐EpOME through the TLR4/Keap1/NRF2 axis, enhancing EMT and metastatic potential [224]. Another CRC‐associated bacterium ETBF increased the expression of JmjC‐domain containing histone demethylase 2B (JMJD2B) via the TLR4–NFAT5 pathway, thereby enhancing tumor stem cell properties [225]. In skin cancer, commensal cutaneous microbiota activates TLR5, triggering proinflammatory responses that promote tumorigenesis [226]. In the pancreatic cancer models, cancer‐associated microbiota induces an immunosuppressive phenotype in macrophages through TLR signaling, limiting T cell‐mediated immune responses [10]. Interestingly, certain microbiota also exert antitumor effects via TLR pathways. For example, flagellar protein B secreted by *Salmonella typhimurium* activates TLR signaling, promoting M1 macrophage polarization and enhancing antitumor immune responses [227]. Additionally, *Hominenteromicrobium*, isolated from fecal samples of PD‐1 inhibitor responders, activates S6K and STAT3 phosphorylation through the TLR7/9–MyD88 signaling axis, upregulating IRF8 expression to promote classical dendritic cell differentiation. This enhances tumor‐specific CD8^+^ T cell activation and improves the efficacy of PD‐1 blockade [228].

It should be noted, however, that TLR4 signaling outcomes are not uniform across tissues, immune contexts, or host species. TLR4 polymorphisms remodel the LPS‐binding interface, shifting the activation threshold of MyD88/TRIF signaling [229, 230]. This recalibrates downstream NF‐κB/IRF3 kinetics and cytokine output in a context‐dependent manner [231]. Furthermore, molecular docking analyses have demonstrated that different bacterial species engage TLR4 through structurally distinct binding interfaces. Therefore, the mode of TLR4 engagement and the resulting downstream signaling profile may differ between *F. nucleatum* and other TLR4‐activating bacteria [168]. These factors probably contribute to the variability of *F. nucleatum*–TLR4 signaling observed across tissue microenvironments, immune environment, and experimental model systems, which should be considered when inferring findings between cancer types and model organisms.

#### 9.1.2 NF‐κB Signaling

NF‐κB is a key downstream regulator within the TLR signaling pathway [232]. Its classical activation primarily involves the rapid phosphorylation of IκB proteins by IκB kinase, facilitating the dissociation and release of NF‐κB dimers into the nucleus [233]. Emerging studies have highlighted the role of *F. nucleatum* in regulating tumor progression through various NF‐κB‐dependent mechanisms. The adhesin RadD interacts with CD147 on CRC cell surfaces, initiating a PI3K–AKT–NF‐κB–MMP9 cascade that promotes tumor development [234]. Zhang et al. identified an alternative mechanism by which *F. nucleatum* activates the NF‐κB pathway [235]. Specifically, *F. nucleatum* activated NF‐κB in an ALPK1‐dependent manner to increase ICAM1 expression, promoting extravasation and metastasis of CRC cells. Additionally, *F. nucleatum* induced NF‐κB activation via the NOD1/RIPK2 pathway, facilitating the progression of esophageal squamous cell carcinoma (ESCC) [236]. ETBF secretes BFT, which activates an IL‐17‐dependent NF‐κB inflammatory cascade, triggering the CXCL1–CXCR2 axis to recruit myeloid cells and promoting distal colonic tumorigenesis [22]. Han et al. confirmed the essential role of gut microbiota in mediating zinc finger protein 90 (Zfp90)‐driven progression of colitis‐associated CRC and further indicated that Zfp90‐mediated carcinogenesis involves the activation of NF‐κB [237]. In contrast, *Lactobacillus intestinalis* enhanced antitumor immunity by promoting dendritic cell recruitment via the NOD1/NF‐κB signaling pathway, suggesting that the NF‐κB pathway plays diverse regulatory roles in microbe–host interactions [238].

#### 9.1.3 CGAS–STING Signaling

The cGAS–STING signaling pathway, crucial to the innate immune system, recognizes cytosolic DNA and triggers the expression of IFN‐I and proinflammatory cytokines, exerting immunoregulatory effects [239]. Specific probiotics, including *Lactobacillus rhamnosus* GG and *Bifidobacterium*, induce dendritic cells to produce IFN‐I via the cGAS–STING pathway, enhancing dendritic cell function and promoting cross‐priming of CD8^+^ T cells [240, 241]. Microbiota‐derived STING agonists can stimulate tumor‐infiltrating monocytes to produce IFN‐I, influencing macrophage polarization and modulating interactions between natural killer cells and dendritic cells [23]. Notably, gut dysbiosis impairs effector T cell function by suppressing the cGAS–STING pathway, while the bacterial second messenger c‐di‐AMP enhances antigen presentation by dendritic cells [242]. However, certain pathogens inhibit the cGAS–STING signaling [242]. Notably, HPV16 E7 disrupts STING stability by hijacking the PRR member NLRX1, thereby evading immune surveillance [243].

#### 9.1.4 Β‐Catenin Signaling

β‐Catenin, a core effector molecule in the Wnt signaling pathway, can be aberrantly activated through dysregulation of the classical Wnt/β‐catenin pathway, impaired degradation of β‐catenin, and enhanced nuclear translocation [244]. This results in sustained activation of downstream TCF/LEF transcription complexes, driving the expression of target genes that promote cancer stem cell renewal, cell proliferation, and differentiation, thereby facilitating tumorigenesis and progression. Recent studies have highlighted the regulatory role of the microbiota in tumors via the β‐catenin signaling pathway. The BFT toxin secreted by ETBF not only inhibits the phosphorylation and degradation of β‐catenin but also promotes its synergistic interaction with the Notch pathway, accelerating stemness and metastasis in breast cancer [245]. Similarly, Rubinstein and colleagues found that *F. nucleatum* induced E‐cadherin phosphorylation and endocytosis via its surface adhesin FadA, facilitating β‐catenin release and nuclear translocation, directly promoting CRC [246]. They also indicated that this mechanism operates independently of inflammation induced by bacterial internalization. Further investigations revealed that the *Salmonella* Type III secretion effector AvrA functions as a deubiquitinating enzyme, suppressing K48‐linked ubiquitination and degradation of β‐catenin. Additionally, AvrA activates Akt kinase to promote Ser552 phosphorylation, facilitating β‐catenin nuclear translocation and inducing colonic tumorigenesis [247, 248]. Notably, the microbiota's regulatory role is not always tumor promoting. A recent study demonstrated that psychological stress‐induced gut dysbiosis reduced *Akkermansia muciniphila* and its metabolite, butyrate, thereby enhancing breast cancer cell stemness [249]. Mechanistically, butyrate upregulated the expression of RNA‐binding protein ZFP36 by increasing histone H3K9 acetylation, leading to the degradation of LRP5 mRNA and indirectly inhibiting β‐catenin pathway activity. Additionally, *Bifidobacterium adolescentis* specifically activated the Wnt/β‐catenin pathway in CD143^+^ cancer‐associated fibroblasts, inducing the expression of growth arrest‐specific 1 (GAS1) to inhibit CRC, revealing a tumor‐suppressive role of β‐catenin signaling under certain conditions [250]. Bifidobacterium‐derived SCFAs suppress Wnt/β‐catenin signaling in tumor cells through HDAC inhibition and transcriptional repression, reducing CRC proliferation and stemness [194]. Regarding viruses, pathogens interfere with β‐catenin homeostasis through complex mechanisms [251]. Latent membrane proteins (LMP1‐2) of EBV, the primary oncoproteins, regulate the stabilization and nuclear translocation of β‐catenin [252, 253, 254]. HPV E6/E7 oncoproteins synergistically promote β‐catenin nuclear translocation by suppressing its ubiquitination and degradation [255, 256]. HPV E6 protein enhances β‐catenin stabilization and nuclear translocation by binding its PDZ domain, promoting the expression of forkhead box M1 [257, 258, 259]. In contrast, infection with human cytomegalovirus leads to decreased β‐catenin levels in certain cell types, such as fibroblasts and trophoblast cells [260].

#### 9.1.5 ERK Signaling

As a critical signaling pathway within the MAPK cascade, ERK signaling plays a central role in the progression of various cancers by regulating cellular proliferation, differentiation, migration, and survival [261]. Specific pathogens can directly activate this pathway through their virulence factors to drive tumorigenesis. For example, *cagA*‐positive *H. pylori* injects the *cagA* protein into B cells via its Type IV secretion system. Upon phosphorylation, *cagA* binds to intracellular signaling molecules containing SH2 domains, activating the ERK/p38–MAPK cascade. This process upregulates the antiapoptotic proteins Bcl‐2 and Bcl‐xL, inhibiting B cell apoptosis and promoting abnormal proliferation, ultimately contributing to the formation of mucosa‐associated lymphoid tissue (MALT) lymphoma [262]. Importantly, ERK pathway activation by microbial virulence factors is not restricted to *H. pylori*. Beyond H. pylori, multiple bacterial species have been shown to modulate ERK signaling in a tissue‐ and context‐dependent manner. Mu et al. also demonstrated that intracellular *P. gingivalis* activates ERK signaling by secreting gingipain proteins, leading to ERK phosphorylation, AP‐1 transcription factor activation, and S‐phase cell cycle arrest in CRC cells, thus promoting cancer growth [263]. In contrast, beneficial microorganisms can exhibit opposing regulatory effects. For example, oral administration of *Bifidobacterium* has been shown to significantly inhibit glioma growth by suppressing the MEK/ERK axis [264].

#### 9.1.6 JAK–STAT Signaling

The JAK–STAT signaling pathway, an evolutionarily conserved transmembrane pathway, is activated by extracellular signals such as cytokines, IFNs, and growth factors [265]. Microbiota play a significant role in tumorigenesis by mediating the activation of JAK–STAT signaling [74]. An early study revealed that *P. gingivalis* directly activates the STAT3 signaling axis to inhibit apoptosis in epithelial cells, thereby supporting bacterial survival [266]. Further investigation showed that *P. gingivalis* induces the expression of microRNA‐203, which inhibits the key negative regulator SOCS3, leading to sustained STAT3 activation [267]. This sustained activation of JAK–STAT signaling not only facilitates bacterial immune evasion but also promotes tumorigenesis through antiapoptotic effects and uncontrolled proliferation [266, 267]. Notably, the JAK–STAT pathway is involved in regulating Th1/Th2 differentiation [268]. In ovarian cancer tissues, Li et al. observed that host genes associated with microbiota dysbiosis were enriched in immune‐related pathways, including JAK–STAT signaling and Th1/Th2 cell differentiation [269]. However, this study did not explore whether microbiota regulate Th1/Th2 immune imbalance through modulation of JAK–STAT signaling.

#### 9.1.7 PI3K–AKT Signaling

**FIGURE 3:**
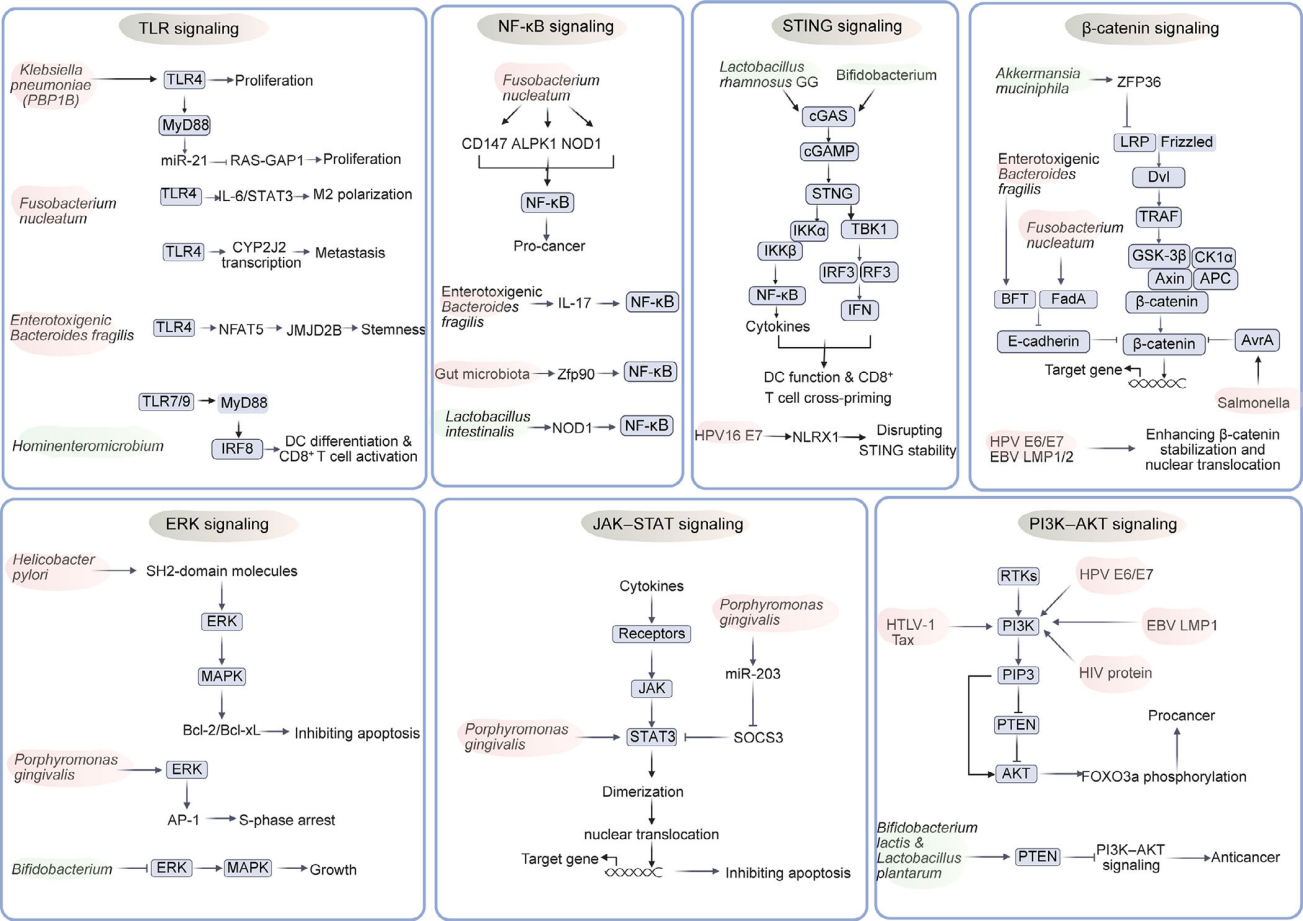
Microbial regulation of innate immune and tumorigenic signaling pathways. Microbial regulation of innate immune and tumorigenic signaling networks. Pathogenic and beneficial microorganisms differentially engage TLR–NF‐κB, cGAS–STING, Wnt/β‐catenin, ERK, JAK–STAT, and PI3K–AKT pathways to shape inflammation, antigen presentation, cell survival, and antitumor immunity. Pathogenic microbes are labeled in red, while beneficial microbes are in green.

Microbiota play a critical role in the dysregulation of the PI3K–Akt signaling pathway, influencing cancer initiation and progression [221]. For instance, the Tax protein encoded by human T‐cell leukemia virus Type I (HTLV‐1) activates PI3K–Akt signaling, leading to the phosphorylation of the tumor suppressor FOXO3a at Thr32 and Ser253 by AKT [270]. This phosphorylation causes FOXO3a to be translocated out of the nucleus, preventing the expression of target genes such as BIM and p130 [271]. As a result, the abnormal activation and proliferation of CD4^+^ T cells infected with HTLV‐1 contribute to the onset and development of adult T‐cell leukemia [270]. Similarly, the E6/E7 oncoproteins of HPV, the LMP1 protein of EBV, and structural proteins of Human immunodeficiency virus (HIV) all activate the PI3K–Akt axis, promoting cancer cell proliferation, acquisition of stem cell characteristics, and invasive capabilities [272, 273, 274, 275]. Tsay et al. discovered that the lower respiratory tract of lung cancer patients is enriched with oral bacterial communities such as *Veillonella*, *Streptococcus*, and *Prevotella* [276]. The enrichment of these oral commensal bacteria was significantly correlated with upregulation of the PI3K–Akt signaling pathway, suggesting that microbiota translocation may influence lung cancer by regulating oncogenic signaling. In gliomas, the loss or inactivation of the tumor suppressor gene PTEN results in sustained PI3K–Akt pathway activation [277, 278]. The combination of *Bifidobacterium lactis* and *Lactobacillus plantarum* enhances PTEN expression, inhibiting the PI3K–Akt signaling pathway and exerting a tumor‐suppressing effect in gliomas (Figure 3 and Table 1) [279].

**TABLE 1:** Microbial modulation of key signaling pathways in cancer.

Microorganism	Receptor	Downstream cascade	Result	Cancer type	Effect	References
*Fusobacterium nucleatum*	TLR4	MyD88→NF‐κB→miR‐21 expression→RASA1 inhibition	Promote the proliferation of cancer cells	CRC	Procancer	[183]
*Klebsiella pneumoniae*	TLR4	MyD88→NF‐κB→oncogene expression	Promote the proliferation of cancer cells	HCC	Procancer	[222]
*Fusobacterium nucleatum*	TLR4	IL‐6→p‐STAT3→c‐MYC	Promote the immunosuppressive M2 polarization of macrophages	CRC	Procancer	[223]
*Fusobacterium nucleatum*	TLR4	AKT→Keap1 downregulation→NRF2 upregulation→CYP2J2 transcription	Promote the invasion and migration of cancer cells	CRC	Procancer	[224]
Enterotoxigeni*c Bacteroides fragilis*	TLR4	NFAT5→JMJD2B expression→H3K9me3 demethylation→NANOG expression	Promote the stemness of cancer cells	CRC	Procancer	[225]
*Hominenteromicrobium*	TLR7/9	MyD88→S6K/STAT3 phosphorylation→IRF8 expression	Promote the differentiation of cDC cells	Multiple cancers	Anticancer	[228]
*Fusobacterium nucleatum*	CD147	PI3K‐AKT→NF‐κB→MMP9	Promote the invasion and migration of cancer cells	CRC	Procancer	[234]
*Fusobacterium nucleatum*	ALPK1	NF‐κB→ICAM1 transcription	Promote the metastasis of cancer cells	CRC	Procancer	[235]
*Fusobacterium nucleatum*	NOD1	RIPK2→NF‐κB→oncogene expression	Promote the growth of cancer cells	ESCC	Procancer	[236]
*Lactobacillus*	NOD1	NF‐κB→CCL5 expression	Promote the recruitment of dendritic cells	CRC	Anticancer	[238]
*Bifidobacterium*	/	cGAS–STING→IFNβ expression	Promote the cross‐priming of dendritic cells	Multiple cancers	Anticancer	[240]
*Lactobacillus rhamnosus* GG	/	cGAS–STING→TBK1→IRF7→IFNβ expression	Promote the cross‐priming of dendritic cells	Multiple cancers	Anticancer	[241]
Enterotoxigenic *Bacteroides fragilis*	β‐Catenin	β‐catenin nuclear translocation→target gene expression	Promote the proliferation of cancer cells	Breast cancer	Procancer	[245]
*Fusobacterium nucleatum*	β‐Catenin	β‐catenin nuclear translocation→target gene expression	Promote the growth of cancer cells	CRC	Procancer	[246]
*Salmonella*	β‐Catenin	β‐catenin nuclear translocation→target gene expression	Promote the occurrence of cancer cells	CRC	Procancer	[247]
*Akkermansia muciniphila*	LRP5	GSK3β Ser9 phosphorylation→β‐catenin nuclear translocation→target gene expression	Promote the stemness of cancer cells	Breast cancer	Procancer	[249]
*Bifidobacterium adolescentis*	/	Wnt/β‐catenin→TCF4 expression→GAS1 transcription	Promote the formation of cancer‐associated fibroblasts	CRC	Anticancer	[250]
*Helicobacter pylori*	SHP‐2	ERK/MAPK phosphorylation→Bcl‐2/Bcl‐xL expression	Promote the malignant proliferation of B cells	MALT lymphoma	Procancer	[262]
*Porphyromonas gingivalis*	/	ERK/MAPK phosphorylation→AP‐1 transcription	Promote the growth of cancer cells	CRC	Procancer	[263]
*Porphyromonas gingivalis*	/	miR‐203 expression→SOCS3 inhibition→Stat3 phosphorylation→IL‐6 expression	Promote the inflammation of periodontal tissues	Oral cancer	Procancer	[267]

#### 9.1.8 | Others

*Streptococcus thermophilus* has been shown to significantly suppress CRC cell proliferation in vitro [280]. Mechanistically, β‐galactosidase secreted by *S. thermophilus* disrupts glucose uptake and activates AMPK, which subsequently phosphorylates YAP, thereby inhibiting the Hippo pathway. Tumor‐resident microbiota can activate the RhoA–ROCK pathway, remodeling the actin cytoskeleton to support distant metastasis and colonization of breast cancer cells [75]. In epithelial ovarian cancer, dysbiosis enhances the expression of Sonic Hedgehog by triggering the TLR4/NF‐κB signaling pathway, which ultimately activates the Hedgehog signaling and drives cancer progression [281].

### 9.2 Amino Acid Metabolism

#### 9.2.1 Tryptophan

Various microorganisms are capable of metabolizing tryptophan to produce indole compounds, a process observed particularly in members of *Lactobacillus*, *Parabacteroides*, *Bifidobacterium*, *Bacteroides*, *Clostridium*, and *Peptostreptococcus* [282, 283]. Tryptophan is metabolized by aromatic amino acid aminotransferase or tryptophan 2‐monooxygenase into indole‐3‐pyruvate (IPyA) and indole‐3‐acetamide, respectively, which then generate key indole metabolites involved in various metabolic pathways. By activating the aryl hydrocarbon receptor (AhR), indole metabolites can directly regulate cancer cell fate or mediate immune responses that indirectly influence cancer progression [284].

Reduced levels of indole‐3‐lactic acid (ILA) have been associated with the malignancy of CRC, and exogenous ILA treatment has been shown to inhibit cancer cell growth [285]. One study demonstrated that ILA produced by *Lactobacillus gallinarum* selectively induced apoptosis in CRC cells through AhR activation, without affecting colonic epithelial cells [286]. However, Li et al. found that ILA derived from *Bifidobacterium breve* mediated macrophage differentiation and maturation via the AhR/p‐AKT/IL‐1β pathway. Interestingly, ILA failed to trigger AKT phosphorylation in CRC cells [287]. These findings suggest that AhR activation by ILA might be cell‐type specific, but the exact mechanisms remain unclear. While many studies have focused on tumor regulation via ILA–AhR signaling, ILA can also exert anticancer effects independent of AhR. Zhou et al. reported that ILA reprogrammed glucose metabolism by inhibiting STAT3 phosphorylation and reducing hexokinase 2 expression [285]. Additionally, ILA promoted IL‐12 secretion by inducing epigenetic modifications in dendritic cells and suppressed cholesterol metabolism by inhibiting Saa3 in CD8^+^ T cells, collectively enhancing CD8^+^ T cell activation and antitumor responses [288]. ILA also suppressed CRC by targeting the nuclear receptor RORγt, reducing Th17 cell differentiation and impairing the IL‐17 signaling pathway [289].

**FIGURE 4:**
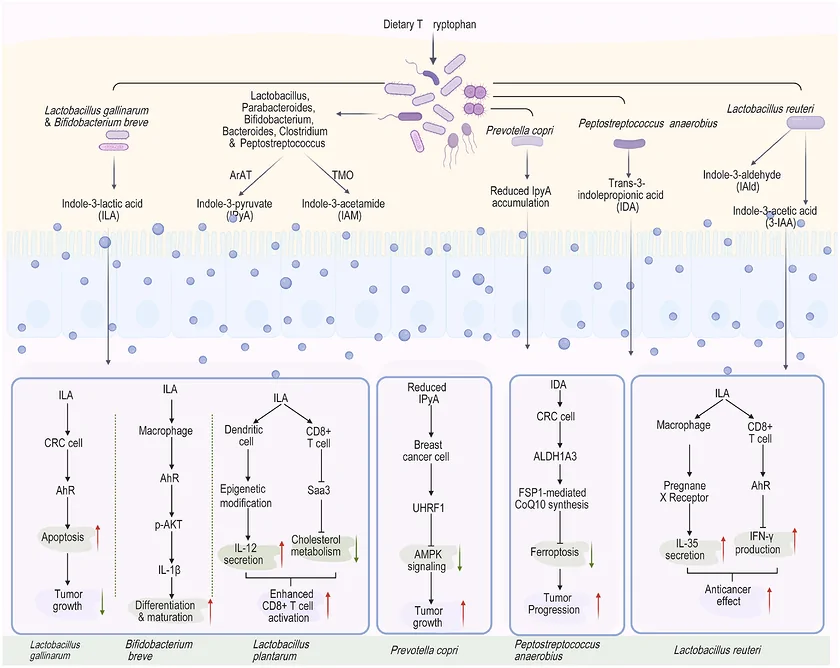
Tryptophan‐derived microbial metabolites regulate cancer through receptor and metabolic pathways. Indole derivatives modulate CRC progression through AhR‐dependent and AhR‐independent pathways. Key regulatory mechanisms include tumor‐cell apoptosis, immune cell regulation (macrophage polarization, dendritic cell function), and metabolic reprogramming.

*Prevotella copri* enrichment promoted breast cancer development by consuming tryptophan, limiting the accumulation of IPyA [290]. The depletion of IPyA enhanced UHRF1‐driven suppression of the AMPK signaling pathway, ultimately promoting tumor growth. *Peptostreptococcus anaerobius*‐derived trans‐3‐indolepropionic acid (IDA) enhanced FSP1‐mediated coenzyme Q10 synthesis by upregulating ALDH1A3, inhibiting ferroptosis and promoting CRC progression [291]. In CRC and melanoma, *Lactobacillus reuteri* synthesized indole‐3‐aldehyde from tryptophan [158, 292]. Indole‐3‐acetic acid (3‐IAA) inhibited tumorigenesis in colitis‐induced CRC by activating the pregnane X receptor on immune cells, particularly macrophages, to induce IL‐35 secretion [292]. Additionally, selective activation of AhR on CD8^+^ T cells resulted in the stimulation of IFN‐γ‐producing CD8^+^ T cells, mediating anticancer effects (Figure 4).

#### 9.2.2 Arginine

Tumor cells and myeloid‐derived suppressor cells highly express arginase 1, which breaks down extracellular arginine, leading to suppressed T cell receptor signaling, G0–G1 arrest, and downregulation of key effector molecules [293, 294]. Low arginine conditions can also induce immunosuppressive Treg cell‐like properties in activated CD4^+^ T cells through the ATF4–SLC7A11–GSH axis [295]. Microorganisms influence arginine metabolism to regulate the tumor immune microenvironment, ultimately determining tumor growth or clearance [296]. Emerging studies suggest that microbial consumption of arginine drives immunosuppression. For example, *Proteobacteria*, most notably *E. coli*, enhance Treg cell function and suppress CD8^+^ T cell activity by degrading arginine, thereby promoting uncontrolled tumor growth in the omentum. Specifically, arginine depletion enhances Treg cell function by inhibiting the mTOR signaling pathway, while arginine supplementation reverses this effect [297]. Similarly, *Streptococcus anginosus* converts arginine into ornithine and polyamines via its arginine deiminase, directly promoting GC cell proliferation and suppressing CD8^+^ T cell anticancer function [298]. Engineered *E. coli* Nissle 1917 colonizes tumors and continuously converts the metabolic waste product ammonia into l‐arginine, elevating intratumoral arginine concentrations and reversing these inhibitory signals to restore T cell proliferation and effector function [299]. A recent study further demonstrates that engineered *E. coli*, which integrate an arginine–nitric oxide synthesis circuit, support T cell metabolism through arginine supplementation [300]. However, increased arginine levels can also exert cancer‐promoting effects in certain tumors. Xu et al. demonstrated that arginine accumulation activated nitric oxide synthesis and polyamine metabolism pathways, promoting tumor progression by inducing M2 macrophage polarization and activating the Wnt/β‐catenin pathway [301].

#### 9.2.3 Bile Acid Metabolism

Bile acids are lipid metabolites derived from cholesterol in the liver, primarily responsible for emulsifying, digesting, and absorbing fats. Primary bile acids synthesized in the liver are secreted into the gut, where gut microbiota biotransform them into secondary bile acids, including deoxycholic acid (DCA) and lithocholic acid (LCA) [302]. Because secondary bile acids are rapidly produced from primary forms and both pools are continuously exchanged via the enterohepatic circulation, hepatic and microbial contributions form an inseparable functional network that is difficult to disentangle quantitatively. Cross‐tissue multiomics analyses have shown that the absence of gut microbiota results in profound immune abnormalities across organs and dramatic shifts in the bile acid composition [303]. It provides direct evidence that microbial transformation is a significant determinant of the bile acid–immune axis. Most existing studies, however, focus on the functional effects of individual bile acid species in specific settings, and quantitative comparisons of the relative contributions of liver‐derived versus microbiota‐derived bile acids remain lacking. Activation of the TGR5 receptor by LCA inhibits breast cancer cell proliferation, reverses EMT, and enhances anticancer immunity [304]. Gut microbiota producing bile salt hydrolases can suppress HCC growth by generating conjugated DCA [305]. However, it is reported that dysregulated bile acid metabolism promotes tumor progression. Cao et al. found that DCA induced intestinal carcinogenesis by promoting gut dysbiosis, disrupting the intestinal barrier, and recruiting M2 macrophages [306]. Similarly, postcholecystectomy dysbiosis increased tauroursodeoxycholic acid production, exacerbating colorectal tumorigenesis by inhibiting the FXR signaling pathway and disrupting FXR/β‐catenin interactions [307]. The immunomodulatory effects of the microbiota‐bile acid axis are also significant. *Akkermansia muciniphila* modulates bile acid metabolism, increasing tauroursodeoxycholic acid levels, which suppresses PD‐L1 expression and enhances immunotherapy efficacy in HCC [308]. Ma et al. demonstrated that gut bacteria‐mediated conversion of primary to secondary bile acids controls CXCL16 (CXC‐chemokine ligand 16) expression on liver sinusoidal endothelial cells, thereby regulating hepatic CXCR6^+^ natural killer T cell recruitment and IFN‐γ production [309]. Sirtuin 5 is a metabolic regulator, and its deficiency leads to bile acid overproduction via peroxisomal biosynthetic enzymes. Accumulated bile acids promote M2‐like polarization of tumor‐associated macrophages through nuclear receptor signaling [310]. The expression of the lipid metabolism regulatory enzyme ACSL4 (acyl‐CoA synthetase long‐chain family member 4) in the liver tissues of patients with hepatitis B‐related HCC was significantly upregulated. ACSL4 upregulation elevates bile acid levels and promotes M2‐like polarization, while ACSL4 silencing in vitro and in vivo restores FXR expression and abrogates this polarization [311]. At the T cell level, primary and secondary bile acids accumulated in the liver can respectively inhibit T cell function by inducing oxidative stress and endoplasmic reticulum stress, thereby impeding tumor‐specific T cell responses [312]. Wei et al. further revealed that GPR120 (G protein‐coupled receptor 120)‐mediated FXR suppression downregulated the efflux pump ABCB11, which led to bile acid retention in HCC cells. Aberrant accumulation of bile acids impaired NLRC5 (NOD‐like receptor family CARD domain containing 5)‐dependent MHC‐I antigen presentation and resulted in tumor cells resistant to T cell recognition [313]. Additionally, a detailed cohort analysis of breast cancer revealed that three bacteria (*Hymenobacter*, *Anaerococcus*, and *Collimonas*) were more abundant in patients with lower bile acid metabolism, and this group showed higher levels of M1 macrophage infiltration [314]. However, it was important to emphasize that while these taxa were identified via bioinformatic pipelines in specific cohorts, their presence must be interpreted with caution. Collectively, gut microbiota‐mediated alterations in the tumor immune microenvironment via bile acid metabolism have emerged as a crucial factor influencing tumor burden [315].

#### 9.2.4 Short‐Chain Fatty Acids

**FIGURE 5:**
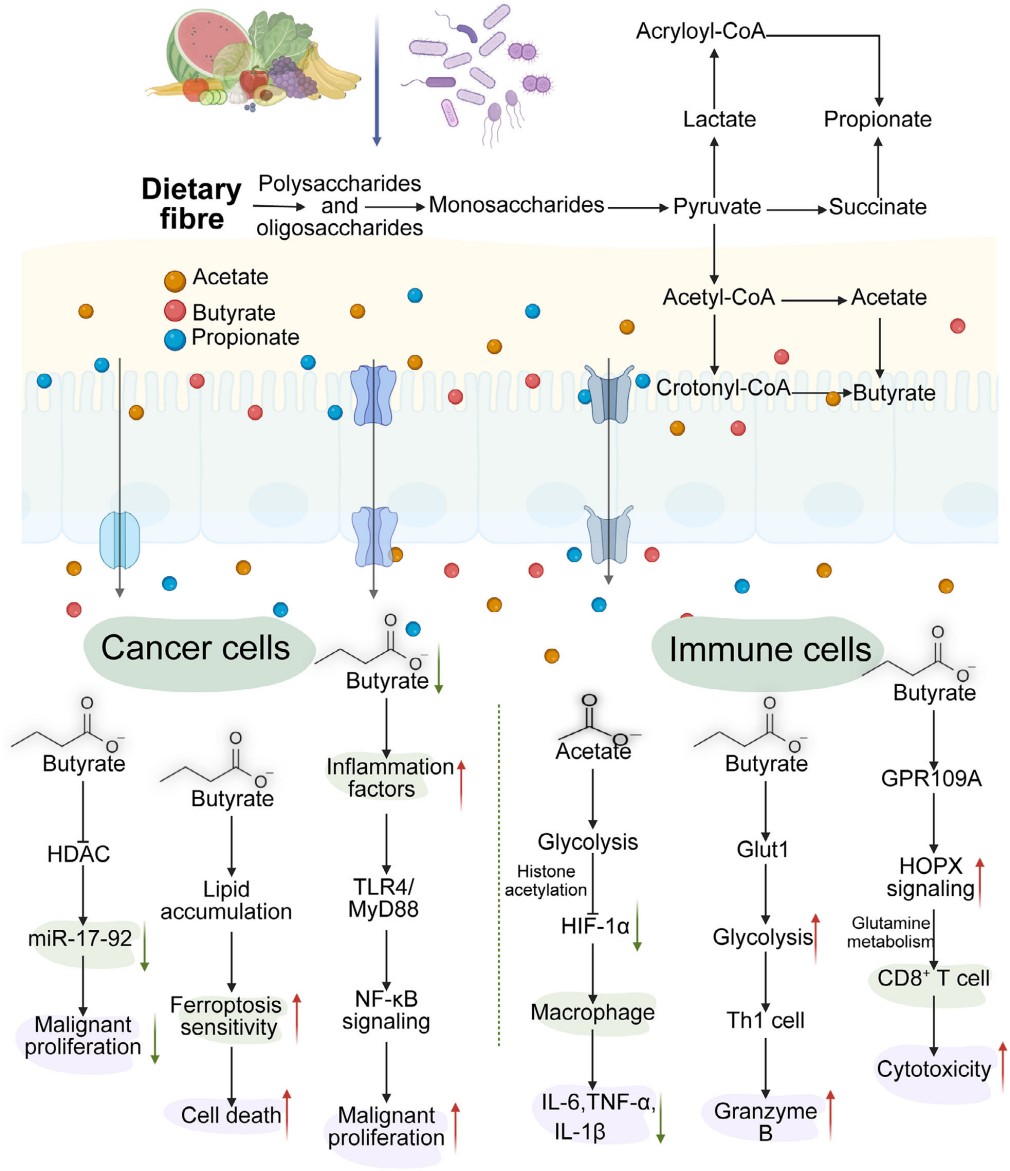
Short‐chain fatty acids link microbial fermentation to tumor metabolism and immunity. Short‐chain fatty acids regulate histone acetylation, lipid metabolism, ferroptosis sensitivity, barrier integrity, inflammatory signaling, and lymphoid and myeloid cell function.

The production of SCFAs from dietary fiber involves a synergistic microbial network, with primary degraders such as *Bifidobacterium* and certain *Clostridium* species breaking down complex polysaccharides, while secondary utilizers like *Escherichia* fermenting the resulting simpler sugars into metabolites like acetic acid [316]. SCFAs, particularly butyrate, influence several cancers by modulating metabolism and immune responses [317]. Butyrate serves as the primary energy source for colonocytes and reinforces intestinal barrier integrity by upregulating tight junction proteins such as claudin‐1 and occludin, thereby reducing paracellular permeability [318, 319, 320]. However, SCFAs can translocate across the epithelium via monocarboxylate transporters (MCT1) and sodium‐coupled monocarboxylate transporters (SMCT1), entering the portal circulation to reach the liver and systemic tissues. Butyrate inhibits HDACs in Treg cells and macrophages, promoting their anti‐inflammatory polarization [321, 322, 323]. Specifically, butyrate‐mediated HDAC inhibition in Treg cells enhances Foxp3 expression and suppressive function [324, 325]. While in macrophages, it shifts polarization from the protumorigenic M2 phenotype toward the antitumorigenic M1 phenotype through STAT6 and NF‐κB pathway modulation [326, 327]. In the context of PDAC, butyrate or its producer, *Clostridium butyricum*, induces oxidative stress and intracellular lipid accumulation, thereby enhancing ferroptosis sensitivity and inhibiting cancer progression [328]. Conversely, butyrate deficiency due to a reduction in *Eubacterium rectum* leads to increased inflammation and elevated TNF levels, resulting in abnormal activation of the TLR4/MyD88/NF‐κB pathway in B cells and promoting malignant B‐cell proliferation. Supplementation with *Eubacterium rectum* reduces TNF levels and lymphoma incidence [329]. In germ‐free mice lacking microbiota, antigen‐activated CD8^+^ T cells fail to efficiently differentiate into long‐lived memory cells. Butyrate acts directly on host CD8+ T cells to decouple their tricarboxylic acid cycle from glycolytic inputs, thereby preferentially driving oxidative phosphorylation and enhancing long‐term memory cell formation (Figure 5 and Table 2) [330].

**TABLE 2:** Microbiota‐mediated metabolic regulation in the tumor microenvironment.

Pathway	Cascade axis	Microorganism	Target site/cell type	Molecular mechanism	Biological effect	Cancer type	References
Tryptophan metabolism	ILA–AhR	*Lactobacillus gallinarum*	CRC cells	Produces indole‐3‐lactic acid (ILA), activates AhR	Selectively induces apoptosis in CRC cells	CRC	[286]
	ILA–AhR/p‐AKT/IL‐1β	*Bifidobacterium breve*	Macrophages	ILA mediates macrophage maturation via AhR/p‐AKT/IL‐1β	Modulates immune environment	CRC	[287]
	IDA/FSP1/CoQ10	*Peptostreptococcus anaerobius*	CRC cells	Produces IDA, upregulates ALDH1A3 to enhances CoQ10 synthesis	Inhibits ferroptosis, promotes CRC progression	CRC	[291]
Arginine metabolism	Not reported	*Streptococcus anginosus*	Gastric cancer cells, CD8^+^ T cells	Converts arginine to ornithine and polyamines	Promotes proliferation, suppresses CD8^+^ T cells	Gastric cancer	[298]
	Not reported	*E. coli* Nissle 1917	Not reported	Synthesizes l‐arginine within tumors, promotes T‐cell infiltration	Suppresses tumor growth	Solid tumors	[299]
Bile acid metabolism	Not reported	Not reported	Breast cancer cells	Lithocholic acid activates TGR5 receptor	Inhibits proliferation, reverses EMT, enhances immunity	Breast cancer	[304]
	FXR/β‐catenin	Not reported	CRC cells	Postcholecystectomy dysbiosis inhibits FXR signaling, disrupts FXR/β‐catenin interaction	Exacerbates colorectal tumorigenesis	CRC	[307]
Short‐chain fatty acids metabolism	TLR4/MyD88/NF‐κB	*Eubacterium rectale*	B cells	Butyrate deficiency induces aberrant TLR4/MyD88/NF‐κB activation	Inhibits malignant B‐cell proliferation	Lymphoma	[329]

The effects of these microbiota‐derived signals, notably SCFAs like butyrate and tryptophan‐derived indoles, are critically defined by a dynamic interplay of cell‐type specificity, receptor context, and local concentration. In healthy colonic epithelial cells, butyrate is efficiently imported and oxidized to acetyl‐CoA, serving both as an energy source and a substrate for histone acetylation, which supports epithelial renewal and barrier integrity [331, 332, 333, 334]. However, within CRC cells exhibiting the Warburg effect, butyrate accumulates and acts as a potent HDAC inhibitor, altering gene expression to arrest the cell cycle [335, 336]. This contrasting phenomenon is named as the butyrate paradox [337]. AhR is frequently overexpressed in many solid tumors, meaning that a metabolite's net effect is substantially shaped by receptor availability and the subsequent signaling cascade [338, 339]. Finally, microbial metabolites exhibit clear dose‐dependency. The high intraluminal concentration of butyrate in the colon (approximately 10–25 mM) activates high‐affinity receptors like GPR109A and inhibits HDACs to maintain intestinal homeostasis [340]. However, its concentration in peripheral circulation is roughly 0.1% of that in the gut, which fundamentally limits its direct efficacy at distant sites.

## 10 Microbiota and Cancer Therapy: Opportunities and Challenges

The discovery that microbiota influence cancer treatment responses has shifted attention from simply describing microbial changes to actively modifying microbial communities. Current strategies range from restoring beneficial microbial functions to selectively removing harmful bacterial populations or engineering microorganisms with therapeutic properties. Additionally, relevant preclinical animal experiments or clinical trials are organized into a summary table to facilitate comparison of translational promise and risk (Table 3).

**TABLE 3:** Representative preclinical and clinical studies of microbiota‐directed cancer interventions.

Intervention	Therapeutic aim	Treatment	Clinical setting	Mechanism	Registration	Phase	References
Fecal microbiota transplantation	Enhance response to ICI	Healthy‐donor FMT + ICI	First‐line NSCLC and melanoma	Reduction of unfavorable baseline taxa and improved ICI activity	NCT04951583	II	[26]
	Overcome ICI resistance	Responder‐derived FMT + anti‐PD‐1	Anti‐PD‐1‐refractory metastatic melanoma	Donor microbiota transfer with increased immune infiltration and tumor immune activation	NCT03353402	I	[27]
	Overcome ICI resistance	Responder‐derived FMT + anti‐PD‐1	Anti‐PD‐1‐refractory advanced melanoma	Microbiome remodeling, increased CD8+ T‐cell activation, and reduced IL‐8+ myeloid cells	NCT03341143	II	[29]
	Enhance response to ICI	Healthy‐donor FMT + anti‐PD‐1	Previously untreated advanced melanoma	Donor‐strain engraftment and enrichment of immunogenic taxa	NCT03772899	I	[341]
	Enhance response to ICI	Responder‐derived FMT + pembrolizumab/axitinib	Previously untreated metastatic renal‐cell carcinoma	Transfer of microbiota from complete ICI responders and donor‐strain engraftment	NCT04758507	IIa	[342]
	Enhance response to ICI	Healthy‐donor FMT + ICI‐based therapy	Previously untreated metastatic renal‐cell carcinoma	Healthy‐donor microbial engraftment during ICI‐based therapy	NCT04163289	I	[343]
	Overcome ICI resistance	Healthy‐donor FMT + anti‐PD‐1	Anti‐PD‐(L)1‐refractory microsatellite‐stable gastrointestinal cancer, mainly gastric cancer	Donor‐microbiota transfer and remodeling of ICI‐resistant gut ecosystems	NCT04130763	I	[344]
Defined microbial products	Enhance response to ICI	SER‐401 + nivolumab	ICI‐naïve metastatic melanoma	Firmicutes‐enriched spore consortium with antibiotic preconditioning	NCT03817125	Ib	[28]
	Enhance response to ICI	CBM588 + nivolumab/ipilimumab	Previously untreated metastatic renal‐cell carcinoma	Live bacterial supplementation and gut microbiota modulation during dual ICI therapy	NCT03829111	I	[345]
	Enhance response to combination therapy	CBM588 + cabozantinib/nivolumab	Advanced or metastatic renal‐cell carcinoma	CBM588‐mediated gut microbiota modulation	NCT05122546	I	[346]
	Enhance response to ICI	MET4 microbial consortium + ICI	Advanced solid tumors receiving ICIs	Defined 30‐strain microbial consortium as a standardized alternative to FMT	NCT03686202	II/III	[347]
Tumor‐targeting bacteria	Direct tumor lysis	*C. novyi*‐NT	Treatment‐refractory advanced solid tumors	Selective germination in hypoxic tumor regions, with direct oncolysis and inflammation	NCT01924689	I	[348]
	Combine bacterial oncolysis with ICI	*C. novyi*‐NT + pembrolizumab	Advanced injectable solid tumors	Hypoxia‐targeted oncolysis combined with PD‐1 blockade	NCT03435952	Ib	[349]

### 10.1 Therapeutic Modulation of Microbiota

#### 10.1.1 Antibiotics

Antibiotics are commonly used to modulate the microbiome, yet their effects on cancer therapies are complex [350]. Metronidazole, for example, reduced the abundance of *Fusobacterium* in CRC models, inhibiting cancer cell proliferation. However, erythromycin‐resistant *Fusobacterium* had no impact on bacterial load or cancer growth, indicating that the effectiveness of antibiotics is closely linked to their ability to clear specific pathogens [351]. Oral or aerosolized ampicillin decreased *Staphylococcus epidermidis* load and suppressed the progression of mammary tumors in mice. Moreover, combining paclitaxel with ampicillin further enhanced breast cancer chemotherapy efficacy [352]. Despite these benefits, systemic antibiotics can disrupt the gut microbiota, negatively affecting cancer treatment outcomes. A systematic review and meta‐analysis examining the impact of antibiotics on patients with NSCLC treated with immune checkpoint inhibitors (ICIs) found that antibiotic use prior to or during ICI therapy disrupted the microbiome, impairing anticancer immune responses and decreasing progression‐free survival (PFS) and overall survival (OS) [353]. In patients receiving anti‐PD‐1/PD‐L1 therapy, antibiotic administration was associated with an increased risk of immune‐related adverse events [354]. A retrospective clinical analysis also showed that antibiotic‐induced gut dysbiosis in mouse models of epithelial ovarian cancer potentially promoted tumor growth and reduced cisplatin sensitivity [355]. Consequently, targeting tumor‐infiltrating microorganisms while preserving gut microbiota integrity presents a significant challenge. The use of membrane‐permeable antibiotics, such as doxycycline, offers a potential strategy for targeting intracellular microbes [75]. Three fundamental challenges underlie this difficulty. Many tumor‐associated bacteria, including *F. nucleatum* and ETBF, are not obligate pathogens but rather commensal members of the healthy human microbiota. Their pathogenicity depends on specific virulence factors, host immune status, and microenvironmental conditions [356]. Conventional antibiotics cannot reliably distinguish these harmful subpopulations from beneficial commensals, causing off‐target damage to the gut ecosystem. Furthermore, tumor‐associated bacteria can invade and persist within tumor cells and immune cells, an intracellular niche that shields them from many antibiotics and host immune clearance [357]. Finally, the gut microbiota functions as a highly interconnected metabolic and immune network, such that targeted elimination of a single species may trigger unpredictable cascading effects through cross‐feeding and metabolic competition [358]. To overcome these challenges, several precision strategies have been developed. Magnetic resonance imaging‐guided targeting employs bacterium‐specific peptide probes to localize tumor‐associated bacteria and then direct high‐dose, low‐toxicity antibiotic delivery exclusively to the tumor site [359]. It effectively reduces the exposure of drugs to healthy tissues. Geng et al. designed a biomimetic nanovehicle for on‐site antibiotic delivery [360]. Biomimetic nanocarriers coated with bacterial proteins or membranes exploit the natural tumor‐homing properties of bacteria to achieve high‐specificity drug delivery with minimal impact on gut microbiota. Enzyme‐responsive prodrugs designed to be activated by macrophage‐specific enzymes can selectively clear intracellular *F. nucleatum* residing within macrophages, thereby reversing immunosuppression and sensitizing tumors to immunotherapy [106]. It was also reported that PEGylated nanoantibiotics effectively eliminated intratumoral bacteria while largely sparing the gut microbiota and simultaneously enhancing therapy efficacy [361]. Specifically, it was produced by modifying antibiotics such as metronidazole with polyethylene glycol, and then self‐assemble into nanoscale particles to preferentially accumulate in tumor tissue through the enhanced permeability and retention effect. Altogether, these approaches represent a shift from broad‐spectrum microbial perturbation to precise, tumor‐confined manipulation, which aim to maximize antitumor efficacy and preserve gut microbial homeostasis.

#### 10.1.2 Fecal Microbiota Transplantation

FMT has shown promise in reshaping the recipient's gut mucosal immunity [362]. Traditional hypotheses suggest that donor strain colonization correlates with clinical success, but Schmidt et al. argue that recipient factors and donor–recipient complementarity are the primary determinants of strain population dynamics [363, 364]. Transplanting microbiota from immunotherapy responders into melanoma‐bearing mice enhanced tumor‐specific immune responses, demonstrating that FMT can transfer anticancer immune capabilities [365, 366]. A Phase I clinical trial (NCT03353402) evaluated whether FMT could induce clinical responses in patients with metastatic melanoma who were resistant to anti‐PD‐1 therapy. Among 10 patients, clinical responses (including partial and complete responses) were observed following FMT reinduction therapy [27]. Additionally, Davar et al. confirmed that FMT reversed acquired resistance to anti‐PD‐1 therapies in advanced melanoma patients [29]. Combination therapy with FMT and pembrolizumab significantly extended both PFS and OS to 14 months in these resistant patients. In another Phase I clinical trial (NCT03772899) involving 20 patients with untreated advanced melanoma, FMT combined with PD‐1 inhibitors such as nivolumab or pembrolizumab demonstrated an objective response rate of 65%, significantly outperforming historical single‐agent data [341]. FMT‐based approaches have also been tested in other tumor types. In metastatic renal‐cell carcinoma, a randomized Phase 2a trial evaluated responder‐derived FMT with pembrolizumab and axitinib. The trial did not meet its primary 12‐month PFS endpoint, but median PFS was longer in the donor‐FMT group than in the placebo‐FMT group (NCT04758507) [342]. Another Phase I study combined healthy‐donor FMT with ICI‐based therapy in metastatic renal‐cell carcinoma and reported acceptable safety, microbial engraftment, and objective responses in some patients (NCT04163289) [343]. In the gastrointestinal cancer, resistant to anti‐PD‐L1 treatment, oral FMT capsules combined with anti‐PD‐1 therapy were also feasible, with early evidence of disease control in some patients (NCT04130763) [344]. These findings broaden the clinical setting for FMT, but the evidence remains early and donor selection is still unresolved. Furthermore, FMT alleviated ICI‐induced colitis [367]. While FMT generally has a favorable safety profile, concerns about the transmission of drug‐resistant bacteria remain and warrant serious attention. Even among apparently healthy donor populations, a study involving 159 subjects has showed that the carriage rate of extended‐spectrum beta‐lactamase (ESBL)‐producing *E. coli* can exceed 35%, representing a substantial transmission reservoir [368]. A landmark safety alert was triggered by two cases in 2019 in which immunocompromised patients acquired bloodstream infections caused by ESBL‐producing *E. coli* linked to the same donor, resulting in one fatality [369]. This event exposed critical drawbacks in then‐current donor screening protocols and prompted global regulatory agencies. United States Food and Drug Administration (US FDA) has issued new safety communications mandating to enhance multidrug‐resistant organism (MDRO) screening. Current US FDA requirements specify that donor stool must be tested for MDRO, including ESBL‐producing Enterobacterales, vancomycin‐resistant enterococci, carbapenem‐resistant Enterobacterales, and methicillin‐resistant Staphylococcus aureus. A defined 60‐day window for repeat testing before and after a donation period has been introduced to ensure dynamic safety. Comprehensive donor screening programs now follow a multitiered workflow that includes an initial questionnaire to exclude individuals with high‐risk medical, travel, or occupational histories, serological testing for infectious disease markers, and stool analysis. Despite these advances, persistent challenges remain. The microbiota of even regularly screened donors can fluctuate dynamically, and non‐MDR strains may still cause infection in susceptible recipients, indicating that screening may need to extend beyond current MDRO targets [370, 371]. These concerns have raised issues regarding the reproducibility and quality control of FMT, given the variability in safety profiles, quantification methods, and practical implementation. To further reduce safety risks, a technique known as washed microbiota transplantation (WMT) has emerged. WMT uses automated washing processes to remove pathogens and metabolites, thereby standardizing microbial suspensions and improving the safety of administration [372, 373]. Collectively, these evolving standards and technologies are essential for realizing the therapeutic potential of FMT while minimizing the risk of drug‐resistant bacterial transmission.

#### 10.1.3 Probiotics, Prebiotics, and Postbiotics

**FIGURE 6:**
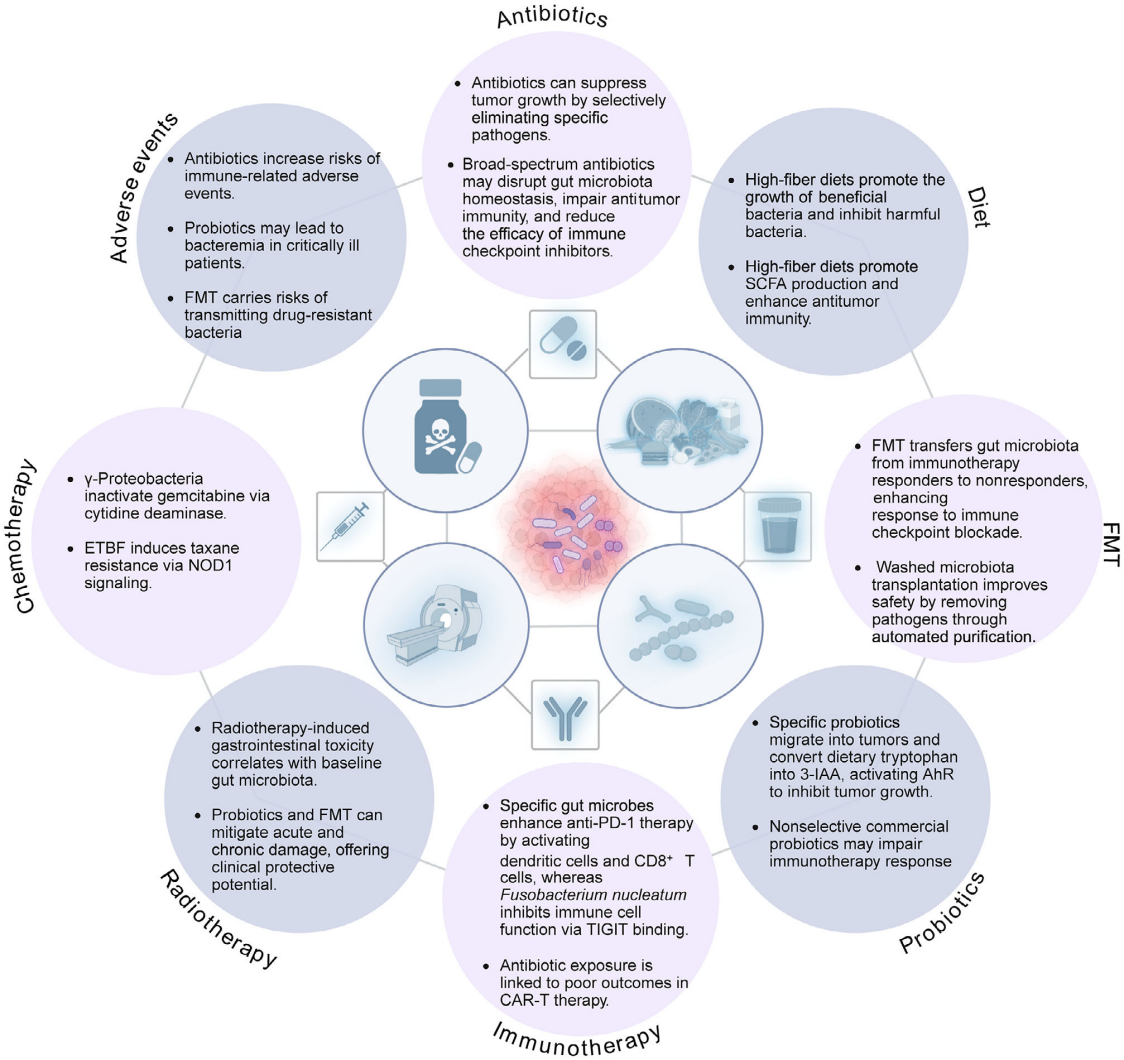
Therapeutic modulation of the microbiota in cancer treatment. Antibiotics, fecal microbiota transplantation, probiotics, and diets can alter treatment efficacy and toxicity, but their clinical effects depend on target specificity, donor or strain selection, baseline community structure, and safety controls.

Probiotics are defined as live microorganisms that are beneficial to the health of the host when given a certain amount. They exert antitumor effects through mechanisms such as immune regulation and metabolic product generation. For instance, the synergistic action of *Lactobacillus johnsonii* and *Clostridium* in producing indole‐3‐propionic acid enhances the efficacy of αPD‐1 immunotherapy, primarily through CD8^+^ T cell‐mediated mechanisms [374]. Following modification with a gallium–polyphenol network, *Lactobacillus rhamnosus* GG significantly reduced the abundance of carcinogenic *Proteobacteria* and microbial LPS levels within pancreatic cancer, thereby inhibiting tumor development [375]. Oral probiotic *Lactobacillus reuteri* migrated to tumor tissues and converted dietary tryptophan into 3‐IAA, inhibiting melanoma growth and prolonging survival in mice. This anticancer effect extended beyond melanoma, also proving effective in CRC and breast cancer models [158]. In metastatic renal‐cell carcinoma, CBM588, a bifidogenic live bacterial product, has been used with nivolumab/ipilimumab (NCT03829111) and with cabozantinib/nivolumab (NCT05122546) [345, 346]. Neither study approached its predefined Bifidobacterium‐enrichment endpoint. However, both reported numerically better clinical outcomes in the CBM588 group, including longer PFS in the nivolumab/ipilimumab study and a higher response rate in the cabozantinib/nivolumab study. MET4, a defined 30‐strain microbial consortium, has also been combined with ICIs in patients with advanced solid tumors, with acceptable tolerability and detectable engraftment of several administered taxa (NCT03686202) [347]. However, probiotics also have obvious limitations. In cancer patients with weakened immune systems, the use of live bacteria poses a risk of opportunistic infection, and the survival rate of probiotics during gastrointestinal transport may be relatively low. Additionally, strain specificity is of crucial importance. Specifically, indiscriminate use of commercial probiotics may undermine immunotherapy efficacy and potentially induce immune‐related adverse events [376]. Commercial probiotics containing *Bifidobacterium longum* or *Lactobacillus rhamnosus* GG were found to hinder the response to ICIs in melanoma models [377]. Prebiotics are nondigestible substrates that selectively stimulate the growth and activity of beneficial indigenous bacteria. Diet plays a crucial role in this context, as high‐fiber diets act as prebiotic sources, correlating with greater microbial diversity and enrichment of beneficial taxa, which potentially improves PFS in patients treated with ICB [377]. Preclinical models have demonstrated the promise of prebiotics in enhancing anticancer immunity and treatment responses in melanoma and anal canal squamous cell cancer (NCT03870607, NCT03950635). The primary disadvantage of prebiotics is that their efficacy relies heavily on the baseline composition of the patient's microbiome; individuals lacking specific bacteria may not derive clinical benefits. Postbiotics are defined as nonliving microbial preparations or their components that are beneficial to the health of the host. Unlike live probiotics, postbiotics offer higher stability at consistent doses and, crucially, higher safety, minimizing the risk of infection in cancer patients with compromised immune systems (Figure 6) [378, 379]. Despite the promising prospects, this field still faces the challenge of standardizing production and dosing protocols.

### 10.2 Microbiome and Response to Cancer Therapies

#### 10.2.1 Immunotherapy

Immunotherapy, including ICIs (such as anti‐CTLA‐4 and anti‐PD‐1/PD‐L1) and chimeric antigen receptor T‐cell (CAR‐T) therapy, is influenced by the microbiome [380]. Moreover, the landmark study by Sivan et al. established that Bifidobacterium enhances dendritic cell function and CD8^+^ T cell priming, supporting its role in potentiating antitumor immunity [366]. In gastrointestinal solid cancers, a clinical trial showed that FMT with specific beneficial microbiota yielded favorable clinical outcomes, with an objective response rate of 7.7% and a disease control rate of 46.2% (NCT04264975). This effect was linked to enhanced cytotoxic T‐cell infiltration and increased T‐cell activity [381]. Transplanting fecal microbiota from responders into germ‐free mice augmented tumor‐infiltrating CD8^+^ T cells, upregulated PD‐L1 expression, and amplified anticancer immunity. In contrast, FMT from nonresponders failed to produce similar effects [382]. Specific microorganisms play a pivotal role in immunotherapy, with *Bacteroidales* linked to the efficacy of CTLA‐4 blockade, while *Bifidobacterium* has been shown to enhance the effect of anti‐PD‐L1 therapy [365, 366]. Additionally, a multicenter cohort study found that the use of high‐risk antibiotics prior to CD19‐targeted CAR‐T therapy was associated with poor prognosis in B‐cell lymphoma [383]. The gut microbiota can enhance antitumor immunity by down‐regulating the PD‐L2–RGMb pathway [384]. Specifically, this pathway is often highly activated in patients who are resistant to PD‐1/PD‐L1 therapy. *Coprobacillus cateniformis* inhibited the activity of this pathway by decreasing PD‐L2 and RGMb expression, suggesting that targeted blockade of the PD‐L2/RGMb interaction could effectively overcome immunotherapy resistance. Moreover, *F. nucleatum* directly suppresses NK and T cell function by binding its Fap2 protein to the T cell immune receptor TIGIT, thereby impairing the immune response [351].

Beyond these direct microbial effects, the intersection of engineered nanomaterials and tumor‐associated bacteria has emerged as a new focus in cancer immunotherapy. Bacteria possess natural tumor tropism, immunostimulatory properties, and programmability, while nanomaterials offer high drug‐loading capacity, tunable release profiles, and multifunctionality [385]. The integration of these two components into nano‐bacterial hybrid systems has established new approaches for precise TME modulation and immune activation in solid tumors [386]. Nanoliposome–bacteria hybrid systems exhibit bacterial chemotaxis to achieve macrophage infiltration and polarization, while expressing CD47 antibodies within the hypoxic TME to enhance its activity [387]. Meanwhile, bacterial outer membrane vesicles (OMVs), serving as natural nanocarriers, can be engineered to deliver immunomodulators such as PD‐1 antagonists, thereby activating antitumor immunity [388]. For instance, bacterial OMVs engineered to display PD‐L1 nanobodies and loaded with cuproptosis‐inducing micelles were developed for triple‐negative breast cancer therapy [389]. This nanoplatform triggered cuproptotic tumor death and blocked immune checkpoint signaling, resulting in markedly enhanced antitumor immune responses characterized by increased CD8^+^ T cell infiltration and activation alongside regulatory T cell depletion. The BROAD‐CAR platform recently reported by Zheng et al. further supports this strategy [390]. By engineering OMVs to express high‐affinity anti‐PD‐L1 antibodies and loading them with plasmids encoding CAR target antigens, BROAD‐CAR enhances CAR‐T antitumor activity through PD‐1/PD‐L1 blockade and promotes intratumoral CAR‐T cell expansion. In a breast cancer mouse model, BROAD‐CAR effectively suppressed tumor recurrence and metastasis. Selenium nanoparticles biosynthesized by *Lactobacillus reuteri* were shown to enhance the in vivo antitumor efficacy of antimesothelin CAR‐T cells in triple‐negative breast cancer models, with gene expression analysis revealing decreased PD‐L1, TGF‐β1, and IL‐10 levels and enhanced proapoptotic signaling [391]. These nanomaterial–microbiota integrations provide a conceptual foundation for next‐generation combination immunotherapies.

#### 10.2.2 Chemotherapy and Radiotherapy

The microbiota plays a significant role in modulating the efficacy of chemotherapeutic agents. In PDAC, γ‐Proteobacteria express the bacterial enzyme cytidine deaminase, which metabolizes the chemotherapeutic drug gemcitabine into an inactive form, reducing its efficacy and conferring drug resistance [392]. *F. nucleatum*, abundant in CRC tissues from patients experiencing cancer recurrence following chemotherapy, induces resistance to oxaliplatin by modulating TLR signaling, microRNAs, and autophagy [393]. 5‐Fluorouracil (5‐FU), a commonly used first‐line chemotherapeutic in CRC, exhibited promising inhibitory effects against *F. nucleatum* in CRC. However, certain bacteria, including *E. coli*, modified 5‐FU and reduced its levels, thereby diminishing its efficacy against *F. nucleatum* and cancer cells [394]. Similarly, etoposide promoted the formation of fluoroquinolone‐resistant *Pseudomonas aeruginosa* biofilms, which protected cancer cells from the cytotoxic effects of the drug [395]. Ma et al. documented that the BFT‐1 toxin from ETBF enhanced breast cancer cell stemness and taxane resistance by activating the NOD1 signaling pathway [13]. Additionally, fungal communities have been shown to negatively influence the efficacy of gemcitabine in PDAC [396]. On the other hand, microorganisms can also enhance chemotherapy outcomes. Cyclophosphamide triggered the translocation of specific Gram‐positive species, including *Lactobacillus johnsonii*, *Lactobacillus murinus*, and *Enterococcus hirae*, from the small intestine to secondary lymphoid organs, where they induced the activation of memory Th1 and pathogenic Th17 cell responses. However, depletion of Gram‐positive bacteria induced tumor resistance to cyclophosphamide, emphasizing the necessity of the microbiota in assisting the drug's antitumor effects [395]. Emerging evidence further highlights the role of specific probiotics in mitigating chemotherapy‐induced toxicity while preserving antitumor efficacy. Capecitabine, an oral prodrug of 5‐FU widely used in CRC, frequently causes intestinal mucositis and diarrhea driven by epithelial damage and dysregulated gut immunity [194]. Preclinical studies demonstrate that *Bifidobacterium infantis* attenuates capecitabine‐induced mucositis by suppressing Th1 and Th17 responses and expanding CD4^+^CD25^+^Foxp3^+^ regulatory T cells in mesenteric lymph nodes, alongside reducing levels of IL‐6, IL‐1β, and TNF‐α [397]. Clinical trials confirm that multistrain probiotics containing *Bifidobacterium longum*, *Bifidobacterium bifidum*, and *Bifidobacterium infantis* safely lower circulating IL‐6, TNF‐α, IL‐17A, and IL‐22 in postoperative CRC patients [398]. Mechanistically, Bifidobacterium engages Toll‐like receptor 2 on intestinal epithelial cells and dendritic cells, activating anti‐inflammatory signaling that promotes IL‐10 production and reinforces gut barrier integrity through SCFAs generation [399]. Species‐specific effects and the direct causal relationships between barrier protection and reduced systemic toxicity warrant further investigation.

Radiotherapy, a treatment effective in destroying tumors, often causes damage to normal tissues, particularly the gastrointestinal tract, resulting in radiation‐related enteritis. The microbiota plays a key role in regulating radiotherapy‐related toxicity, especially the occurrence and severity of radiation‐related enteritis [400]. Microbiome characteristics have shown potential as predictive biomarkers. For instance, patients with lower baseline gut microbial diversity have a higher risk of developing late radiation enteropathy, with increased abundances of *Clostridium IV*, *Roseburia*, and *Phascolarctobacterium* potentially serving as biomarkers for radiotherapy toxicity [401]. Similar predictive value has been observed in patients with prostate cancer and head and neck squamous cell carcinoma [402, 403]. It should be noted, however, that these associations remain largely observational, and direct causal links between microbiota composition and radiation enteropathy are still being established. Radiotherapy dynamically disrupts the microbial community, with animal studies showing increased abundance of *Alistipes* in the large intestine and *Corynebacterium* in the small intestine, alongside an increase in *Bacteroidetes* levels in the colon and feces [404, 405]. Microbiome analysis of cervical cancer patients with acute radiation enteritis has been used to classify the severity of radiation enteritis [406]. Microbiota‐derived metabolites offer protective effects against intestinal radiation injury. Indole‐3‐propionic acid, indole‐3‐carboxaldehyde, and valeric acid attenuate epithelial damage through activation of AhR/IL‐10/Wnt signaling and retention of acyl‐CoA‐binding protein [407, 408, 409]. Additionally, butyrate produced by *Roseburia intestinalis* has been shown to sensitize CRC cells to radiotherapy via the OR51E1/RALB axis, illustrating the therapeutic potential of microbiota modulation to protect normal tissue while enhancing tumor radiosensitivity [410]. Interventions targeting the microbiota have been shown to mitigate radiation‐induced gastrointestinal injury. Prebiotic intake improved stool consistency postradiotherapy, while probiotics reduced damage by modulating immune responses [411, 412]. Notably, FMT has protected against radiation‐induced toxic damage, alleviating symptoms of chronic radiation enteritis, including diarrhea, rectal bleeding, and abdominal discomfort [413, 414, 415]. Beyond these conventional approaches, FLASH radiotherapy delivered at ultra‐high dose rates substantially reduces acute intestinal toxicity, preserves crypt survival, and induces fewer changes to the gut microbiome compared with conventional irradiation [416]. Proton FLASH exposure, in particular, preserves gut commensal microbiomes and intestinal stem cells. This preservation provides a compelling rationale for combining FLASH‐radiotherapy with targeted microbiota modulation, which could therapeutically mitigate radiation enteropathy while maintaining antitumor efficacy [417].

#### 10.2.3 Microbiome‐Related Adverse Events and Toxicity

Microbiome interventions can lead to adverse events. As previously highlighted, antibiotic administration is associated with an increased risk of immune‐related adverse events [354]. Antibiotic exposure alters gut microbiota communities, which correlates with heightened neurotoxicity in cohorts of B‐cell lymphoma and leukemia patients [418]. One study reported a significantly increased risk of *Lactobacillus* bacteremia in patients in the intensive care unit, particularly among those receiving probiotic interventions [419]. Although *Lactobacillus* are generally regarded as safe commensals, their translocation into the bloodstream in critically ill patients serves as a significant clinical marker of severe intestinal barrier dysfunction and extreme host vulnerability. Despite the rareness of these infections, they are often associated with high disease severity scores at baseline, reflecting the compromised state of the host rather than high bacterial virulence alone. Additionally, while FMT generally exhibits a favorable safety profile, it carries the risk of transmitting drug‐resistant bacteria, underscoring the need for stringent quality control (Figure 6) [369].

### 10.3 Microbiota‐Driven Therapeutics

#### 10.3.1 Engineered Bacteria and Microbial Vectors

With the advancement of synthetic biology, several bacterial strains have been engineered into therapeutic platforms with reduced toxicity and enhanced precision in targeting tumor tissues [420]. Attenuated *Salmonella typhimurium* has been used to increase 5‐FU levels within tumors or reshape tumor‐associated macrophage phenotypes by releasing *Vibrio vulnificus* flagellin B [227, 421]. Similarly, the nonpathogenic *E. coli* strain MG1655 induced TNF‐α production in the TME, thereby inhibiting tumor growth [422]. A novel approach enabled the controlled lysis of quorum‐sensing *Salmonella*, where anticancer proteins were produced or released only upon reaching a predetermined population density. This strategy significantly reduced bacterial colony size and mitigated systemic toxicity [423]. In other studies, *E. coli* and *Salmonella* were engineered to lyse synchronously at a threshold population density, periodically releasing chemokines, proapoptotic proteins, or CD47‐targeting nanobody antagonists [424, 425]. Engineered bacteria also show great potential in modulating the tumor immune microenvironment. Oral administration of engineered *E. coli* Nissle 1917, which secreted anti‐PD‐L1 and anti‐CTLA‐4 nanobodies, reduced colonic adenoma burden via immune‐mediated mechanisms [426]. Regarding direct immune activation, attenuated *Listeria monocytogenes* was used to deliver immunogenic tetanus toxoid protein (TT856‐1313) into PDAC, recruiting TT‐specific memory CD4^+^ T cells and inducing tumor killing [427]. *E. coli* Nissle 1917, continuously releasing anti‐PD‐1 and IL‐12, effectively relieved immunosuppression, enhancing the efficacy of ICIs [428]. Additionally, *Salmonella* has been engineered to synthesize IL‐2 or immune checkpoint blockers at the tumor site, activating T cells and reversing T cell exhaustion [429, 430]. Beyond immunomodulation, an attenuated, tumor‐targeting *Salmonella typhimurium* was designed to degrade the extracellular matrix by secreting hyaluronidase, thus enhancing the permeability of chemotherapeutic agents [431].

**FIGURE 7:**
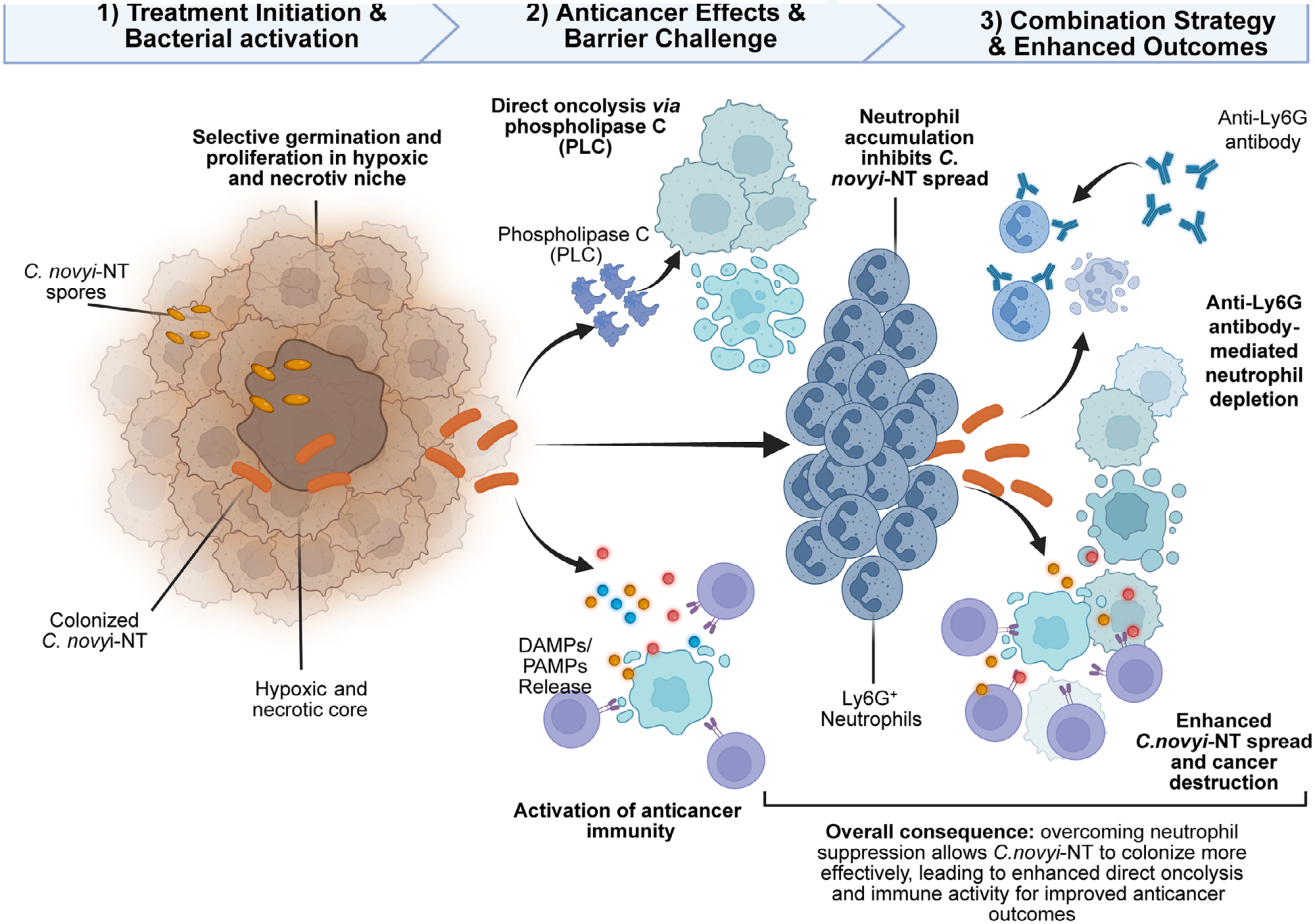
Neutrophil depletion enhances the intratumoral spread and oncolytic activity of *C. novyi*‐NT. *C. novyi*‐NT spores germinate in hypoxic tumor regions and induce phospholipase C‐mediated lysis, whereas Ly6G^+^ neutrophils restrict bacterial dissemination. Anti‐Ly6G treatment removes this barrier and amplifies tumor clearance in animal models.

Clostridium novyi‐NT (C. novyi‐NT), a genetically modified anaerobic bacterium with the toxic α‐toxin gene removed, selectively colonizes and proliferates in the hypoxic and necrotic regions characteristic of solid tumors. Intratumoral injection of C. novyi‐NT spores induces the secretion of phospholipase C, directly lysing cancer cells and activating anticancer immunity [432, 433]. However, the accumulation of neutrophils at the tumor site inhibits the spread of C. novyi‐NT in glioblastoma. Depletion of neutrophils via anti‐Ly6G antibody or hydroxyurea significantly enhanced bacterial dissemination and tumor clearance in both mouse glioblastoma and orthotopic rabbit brain tumor models (Figure 7) [434]. Phase I clinical data confirmed that a single intratumoral injection of C. novyi‐NT in 24 patients with advanced solid cancers achieved a 42% tumor lysis rate at the maximum tolerated dose of 1 × 10^6^ spores (NCT01924689) [348]. A subsequent Phase Ib trial combining C. novyi‐NT with pembrolizumab demonstrated manageable safety in breast cancer and melanoma, supporting synergistic potential (NCT03435952) [349]. Additional strategies to overcome neutrophil‐mediated restriction include CRISPR/Cas9n modification enabling intravenous delivery for pancreatic cancer and combination with irreversible electroporation for colorectal liver metastases [435, 436]. Tumor types with large hypoxic cores, favorable injection accessibility, and controllable neutrophil infiltration, particularly glioblastoma, breast cancer, melanoma, and pancreatic cancer, represent the most promising candidates for C. novyi‐NT therapy.

The application of engineered bacteria has also extended to diagnostics. For instance, modified *E. coli* can report early colonic carcinogenesis or indicate liver metastases by producing urine‐detectable signals [426, 437]. Ho et al. demonstrated that an engineered *E. coli* targeted precancerous cells and converted dietary components in situ into anticancer molecules to suppress CRC development [438]. A bacterial biosensor based on *Acinetobacter baylyi*’s natural ability to ingest exogenous DNA was developed to detect specific DNA mutations related to tumors [439]. Despite these remarkable progresses, clinical translation of live bacterial therapeutics faces various challenges rooted in safety, pharmacokinetics, and interpatient variability. The only completed Phase I trial of attenuated *Salmonella typhimurium* VNP20009 in metastatic melanoma established a maximum tolerated dose of 3 × 10^8^ cfu/m^2^, with dose‐limiting toxicities including thrombocytopenia and bacteremia [440]. However, it observed poor tumor colonization and no objective antitumor responses, sharply illustrating the translational gap between murine models and human patients. This failure underscored that bacterial monotherapy alone cannot simultaneously satisfy biosafety and efficacy, driving the development of second‐generation strains engineered to deliver immunostimulatory payloads such as IL‐15 [441, 442]. Despite dozens of ongoing clinical trials, fundamental challenges still persist in pharmacokinetics, interpatient variability driven by host microbiota and immune status, and lack of predictive preclinical‐to‐clinical scaling models [443]. From a regulatory perspective, US FDA classifies genetically engineered anticancer bacteria as live biotherapeutic products (LBPs). LBPs are regulated by the Center for Biologics Evaluation and Research, which requires following a complete IND (Investigational New Drug Application)‐to‐BLA (Biologics License Application) biologics pathway and meeting stringent CMC (Chemistry, Manufacturing, and Controls) requirements. These requirements encompass strain characterization, genetic stability, biocontainment, and batch consistency [444, 445]. However, no dedicated regulatory guidance for engineered bacteria has yet been issued, creating uncertainty in clinical development. Regulatory pathways for LBPs remain exploratory worldwide. China's Drug Registration Management Measures require strain identification, genetic stability data, in vivo biodistribution studies, and long‐term safety assessments for LBPs [446]. Approved LBPs are targeted in recurrent *Clostridioides difficile* infection, including REBYOTA and VOWST. However, engineered bacteria such as SYNB1891, VNP20009, CRS‐207, and C. novyi‐NT remain at Phase I. Five important barriers hinder regulatory approval, namely, lack of historical precedent, systemic immunotoxicity narrowing the therapeutic window, environmental persistence raising biosafety concerns, unpredictable in vivo behavior due to metabolic complexity, and immature CMC standardization frameworks.

#### 10.3.2 Oncolytic Viruses

Several OVs have been approved for clinical use worldwide, demonstrating their potential for application in cancer treatment. Among these, talimogene laherparepvec, a genetically engineered oncolytic herpes simplex virus (HSV) expressing human granulocyte–macrophage colony‐stimulating factor, has been approved for the treatment of advanced melanoma [447]. H101, approved in China, is an E1B‐deleting adenovirus used for head and neck cancers, as well as EC [448]. Teserpaturev, approved in Japan, is an HSV1‐based mutant for the treatment of malignant glioma [449]. Additionally, the nononcolytic adenovirus Nadofaragene firadenovec, which encodes IFNα‐2b, has recently been approved for bladder cancer treatment [447]. OVs represent a promising therapeutic approach, with mechanisms that extend beyond the direct lysis of infected cancer cells. The interactions between OVs and anticancer immunity have become a focal point of current research [450]. For instance, the oncolytic poxvirus JX‐594 targets various types of cancer cells by activating the EGFR/Ras pathway while suppressing the IFN‐I response [451]. Lymphocytic choriomeningitis virus recruits IFN‐producing Ly6C^+^ monocytes and further activates CD8^+^ T cells, thereby enhancing local anticancer immunity and promoting tumor regression [452]. The combination of OVs with existing immunotherapies is another important strategy for clinical application. OVs can upregulate PD‐L1 expression on both tumor and immune cells, attracting more effector T cells and creating additional sensitive targets for PD‐1/PD‐L1 inhibitors [453, 454]. Furthermore, in triple‐negative breast cancer models, the combination of an oncolytic adenovirus with CAR‐T cells promoted the expression of TGF‐β receptor fusion proteins, enhancing antitumor responses [455]. Through genetic engineering, the functionality of OVs has been further enhanced. For example, researchers have constructed mutant oncolytic vaccinia viruses expressing IL‐2, which successfully reversed immunosuppression by increasing antitumor factor levels and recruiting activated T cells into the TME [456]. Similarly, IL‐7‐loaded oncolytic adenoviruses, combined with B7H3‐targeting CAR‐T cells, prolonged survival in glioblastoma models by providing activating signals to tumor‐infiltrating T cells [457]. However, challenges remain in the clinical translation of OVs, including issues with systemic delivery efficiency, tumor targeting specificity, and potential immunogenicity.

#### 10.3.3 Bacteriophage

Bacteriophages, viruses that specifically target bacteria, have shown unique potential in cancer treatment due to their inherent biological safety, nanoscale regular structure, and suitability for large‐scale production [458]. Current studies on bacteriophage‐based anticancer strategies primarily follows two main directions. One involves using phage display technology to target cancer cells, while the other leverages the natural lytic ability of bacteriophages to eliminate cancer‐associated bacteria.

Phage display technology is a key technique advancing the use of bacteriophages in cancer therapy. Through in vitro or in vivo biopanning processes, it allows for the efficient screening of specific peptides that target tumor cells or associated components [459]. For instance, researchers identified the peptide sequence VTWTPQAWFQWV that selectively bound to glioblastoma cells, and the sequence AGKGTPSLETTP that targeted HCC cells [460, 461]. Thus, bacteriophages are increasingly developed as targeted drug delivery vectors. Zheng et al. demonstrated that covalently linking the chemotherapy drug irinotecan to azide‐modified bacteriophages significantly enhanced its delivery efficiency to CRC tissues [462]. Similarly, coupling doxorubicin with the A54 peptide effectively inhibited tumor growth in HCC models [461]. Beyond chemotherapy, bacteriophages have also been used to deliver photosensitizers or express enzymes locally in tumors to activate prodrugs [463, 464].

However, given that bacteriophages are natural viruses of bacteria, their most immediate and promising application in cancers lies in the precise targeting cancer‐associated bacteria. In CRC, *F. nucleatum* represents a major therapeutic target. The newly isolated lytic phage FNU1 not only clears intracellular *F. nucleatum* but also restores CRC cell sensitivity to 5‐FU and oxaliplatin [465]. Another phage, ØTCUFN3, has been shown to suppress *F. nucleatum*‐induced EMT and inhibit tumor growth in the mouse model [466]. In addition to *F. nucleatum*, advances have also been made against other CRC‐relevant bacteria. For toxigenic *B. fragilis*, precise elimination by bacteriophage VA7 successfully reversed chemotherapy resistance in CRC models [467]. Targeting *Streptococcus gallolyticus*, an early CRC biomarker, a novel DNA nanopatch‐bacteriophage system (DNPs@P) was developed to specifically lyse the bacterium while scavenging intestinal ROS, demonstrating potential for cancer prevention [468]. This antibacterial strategy has been extended to other cancer types as well. Against *H. pylori*, the newly identified lytic phage HPy1R exhibits both acid tolerance and potent antibacterial activity, offering a promising alternative in the face of rising antibiotic resistance [469]. In OSCC, where *P. gingivalis* promotes tumor invasion, an indirect strategy using bacteriophage against its symbiotic partner *Streptococcus gordonii* disrupts biofilm integrity, highlighting the potential of bacteriophage to manipulate complex tumor microbiomes [470].

**FIGURE 8:**
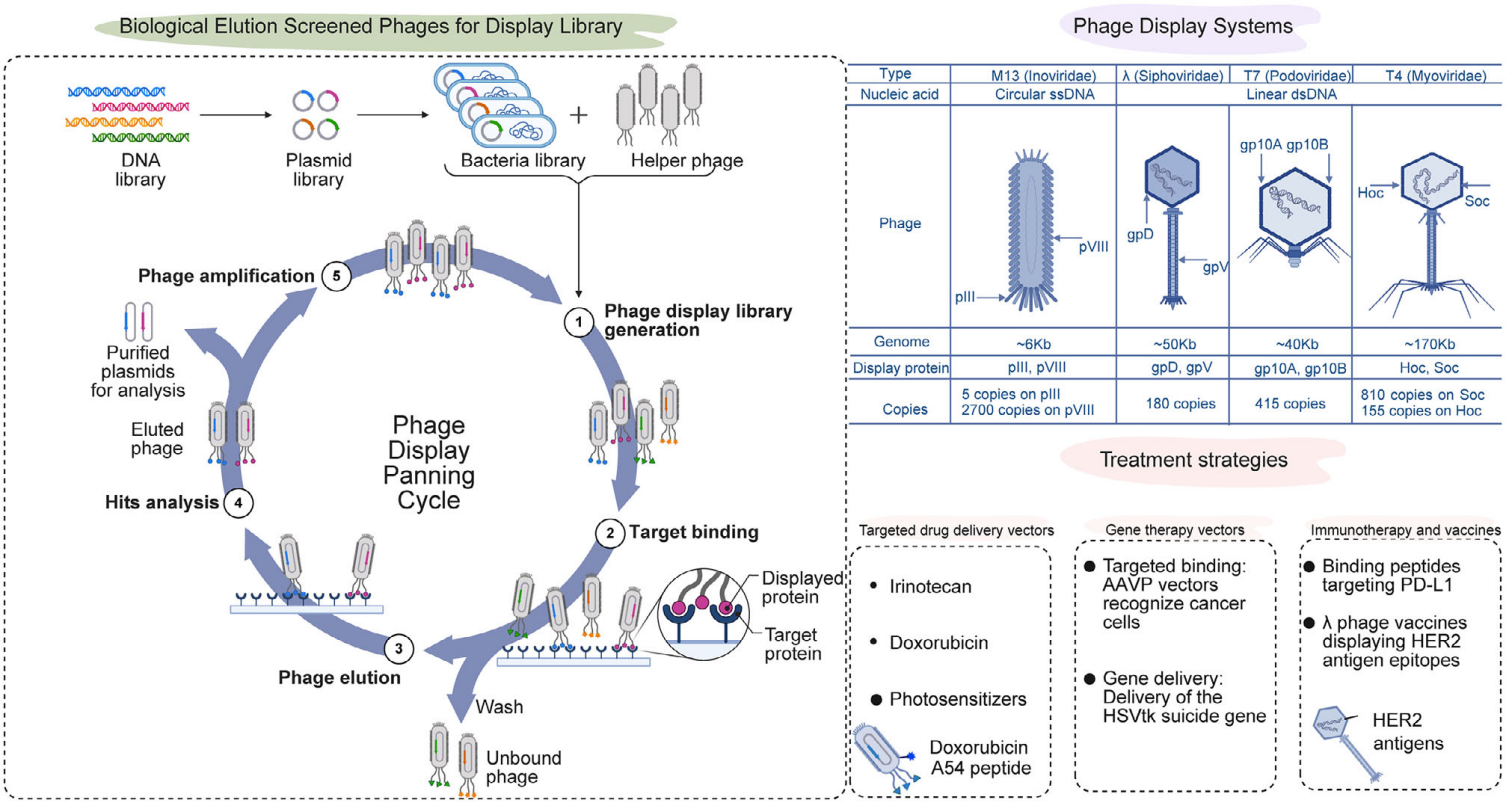
Application of phage display technology in cancer treatment. Bacteriophage platforms for cancer targeting and therapy. M13, λ, T4, and T7 phages support biopanning of tumor‐binding peptides used in phage display systems. Treatment strategies via phage display technology can be adapted as targeted drug delivery vectors, gene therapy vectors, and immunotherapy and vaccines.

Beyond these direct antibacterial applications, bacteriophages continue to serve as versatile vectors in immunotherapy and gene‐associated therapies. In immunotherapy, phage display technology has been used to identify binding peptides targeting PD‐L1, reactivating the anticancer function of T cells [471]. Furthermore, bacteriophages serve as highly effective vaccine platforms [472]. Barati and colleagues demonstrated that λ phage vaccines (λF7), displaying HER2 antigen epitopes, induced a strong immune response mediated by cytotoxic T cells, effectively delaying breast cancer progression [473]. To address the limitations of wild‐type phage gene expression in mammalian cells, a hybrid vector known as adeno‐associated virus and phage (AAVP) has been developed [474, 475]. This vector has been employed to deliver suicide genes such as HSVtk or proapoptotic genes like TRAIL for precise gene therapy (Figure 8) [476, 477, 478]. The value of bacteriophages in cancer treatment extends beyond direct antitumor effects. Integrating the CRISPR–Cas system into bacteriophages targeting *E. coli* has specifically reduced pathogenic strain burden in the mouse intestine, preventing their translocation and bacteremia during cancer treatment [479]. Despite these promising applications, bacteriophage therapy faces challenges. The high specificity of bacteriophages, while advantageous for precise targeting, limits their application, making cocktail therapy involving multiple phages a more reliable strategy [480]. Additionally, immune clearance, vascular barriers, and dense extracellular matrices hinder the penetration and distribution of bacteriophages within tumor tissues (Table 4) [481].

**TABLE 4:** Application of bacteriophages in cancer therapy.

Bacteriaphage	Application type	Core Technology/platform	Mechanism of action	Target or cell type	Payload/expressed product	Model system	Key outcome	Advantages	Limitations and challenges	References
M13 phage	Drug targeted delivery	Phage display	A54 peptide‐mediated targeting accumulation	HCC cells	Doxorubicin‐conjugated A54 peptide	Nude mouse HCC xenograft models	Suppressed tumor growth, prolonged survival	High specificity, low‐dose efficacy	Unknown receptor, residual off‐target toxicity	[461]
P2 phage	Bacterial targeting therapy	Bioorthogonal conjugation	Targeted nanodrug accumulation	Intratumoral *F. nucleatum*	Irinotecan	Mouse and piglet CRC models	Enhanced chemo response, prolonged survival	Oral delivery, tumor‐specific accumulation	Long‐term safety unrevised	[462]
M13 phage	Photodynamic therapy carrier	Phage display	Targeted photosensitizers for localized activation	CRC cells	Rose Bengal photosensitizer	2D and 3D spheroids	Deep penetration and spheroid disruption	High targeting, low‐dose efficacy	Immunogenicity risk	[463]
P22 phage	Enzyme prodrug therapy	Virus‐like particles encapsulation	Encapsulation and intracellular delivery of enzyme	Human cervix carcinoma cells	Cytochrome P450 (CYPBM3)	In vitro HeLa cell transfection	Localized enzyme activation, improved efficacy	Enhanced stability against protease/pH	Immunogenicity and long‐term safety unrevised	[464]
T7 phage	Immune checkpoint blockade	Phage display	Block PD‐1/PD‐L1 interaction via displayed peptides	PD‐L1‐positive tumor cells	PD‐L1‐targeting peptides	In vitro and mouse models	Reactivate antitumor T‐cell function	Enhanced penetration, lower cost	Lower affinity than antibodies	[471]
λ phage	Cancer vaccine platform	Phage display	Induce cytotoxic CD8^+^ T‐cell responses against tumor antigens	HER2‐positive breast cancer cells	HER2 antigenic peptides (AE37)	Mouse breast cancer models	Delay tumor progression, strong immune response	High safety	Clinical translatability unrevised	[473]
M13 phage	Gene therapy vector	RGD4C/AAVP–Grp78	Targeted HSVtk gene delivery	αvβ3 integrin‐positive tumor cells	HSVtk suicide gene	Nude mouse GBM xenograft models	Suppressed tumor growth	Systemic delivery, high specificity	Receptor expression dependent	[476]
M13 phage	Gene therapy vector	Transmorphic phage/AAV	Targeted TRAIL gene delivery	αvβ5‐overexpressing HCC cells	TRAIL suicide gene	In vitro HCC cell transfection	Induced HCC apoptosis	High targeting, low toxicity	Receptor expression dependent	[478]
Tevenvirinae	Infection prophylaxis	Engineered phage, CRISPR–Cas	Specifically lyse *E. coli* strains	Pathogenic Escherichia coli	CRISPR–Cas system	Mice and minipigs	Prevents bacteremia and infection complications	Highly specific, preserves commensal microbiota	Clinical translatability unrevised	[479]

## 11 Diagnostic and Prognostic Potential of the Cancer Microbiome

Beyond its functional roles in tumor initiation, progression, and therapy response, the cancer microbiome has emerged as a potential source of diagnostic and prognostic information. Microbial alterations can reflect changes in tissue environments, immune states, and metabolic conditions associated with tumor development. However, translating microbiome signatures into clinically useful biomarkers requires careful consideration of microbial specificity, biological reproducibility, and methodological consistency.

### 11.1 Microbial Biomarkers for Early Detection

Specific microbial markers are closely linked to the onset and progression of malignancies, highlighting their potential as noninvasive biomarkers [482, 483, 484]. For instance, the enrichment of microbiota such as *F. nucleatum*, *B. fragilis*, and ETBF has been strongly associated with tumor progression in CRC. These microbiota can be reliably detected in stool samples, demonstrating high sensitivity and specificity [485]. Further research has shown that combining fecal microbiome analysis with traditional fecal immunochemical testing (FIT) significantly improves CRC detection, increasing sensitivity from approximately 79% to over 90% compared with FIT alone [486]. This suggests that microbial markers can substantially enhance the effectiveness of existing screening tools rather than merely supplementing them. Additionally, circulating microbial DNA (cmDNA) has emerged as a potential liquid biopsy tool, with cmDNA detected in the plasma of patients with breast cancer, lung cancer, and CRC [487]. The microbiological profile of cmDNA significantly differs from that of healthy controls, supporting its diagnostic potential [488, 489]. Notably, the oral microbiota has been explored for the early detection of pancreatic cancer, with elevated levels of *P. gingivalis* and *Aggregatibacter actinomycetemcomitans* in saliva being associated with PDAC. This suggests that the oral‐gut axis may provide a novel pathway for noninvasive detection [483].

### 11.2 Prognostic Microbiota in Survival and Therapy Response

Microbiota serve as prognostic tools for predicting outcomes and therapy responses in cancer patients. In gastrointestinal cancers, the enrichment of *F. nucleatum* often indicates poor prognosis and is linked to higher mortality and recurrence risk in CRC [351, 490]. High levels of *F. nucleatum* are also associated with reduced survival in GC and poor response to neoadjuvant chemotherapy in ESCC [491, 492]. However, *F. nucleatum* has shown protective effects in OSCC and anal canal squamous cell carcinoma, suggesting a favorable prognosis in these cases [493, 494]. A retrospective cohort study of 802 patients with nasopharyngeal carcinoma found that patients with a high microbial burden had lower disease‐free survival (DFS), distant metastasis‐free survival, and OS compared with those with a low microbial burden [159]. Beyond individual species, specific microbial combinations also demonstrate strong predictive capabilities. *Pseudoxanthomonas*, *Streptomyces*, *Saccharopolyspora*, and *Bacillus clausii* are enriched in long‐term survivors of PDAC, while *Clostridia* and *Bacteroidia* dominate in short‐term survivors [495]. Although *F. nucleatum* is generally linked to poor prognosis, a microbial community comprising *Fusobacterium*, *Granulicatella*, and *Gemella* was independently associated with longer DFS [496]. In lung cancer, *Actinomycetales* and *Pseudomonadales* are correlated with reduced DFS, whereas the presence of *Serratia marcescens*, *Acinetobacter jungii*, and *Streptococcus constellatus* is linked to favorable survival outcomes [497, 498]. Additionally, *Akkermansia muciniphila* is associated with improved immunotherapy responses and survival in both NSCLC and renal cell carcinoma, while *Clostridium* is enriched in the intratumoral microbiome of melanoma tissues among patients who respond to immunotherapy [11, 499, 500].

### 11.3 | Machine Learning and Microbiome‐Based Classifiers

Machine learning, a powerful tool for analyzing the complex relationships between microbiota and cancer, is capable of identifying microbial signatures associated with disease states from large datasets [501]. Advancements in high‐throughput sequencing technologies and computational methods support the development of microbiome‐based classifiers. For instance, the TOPOSCORE classifier predicts patient responses to ICIs by assessing the relative ratios of putative beneficial and detrimental bacteria [502]. Support vector machines have been employed not only for sample classification into different cohorts but also for identifying potential diagnostic or therapeutic targets [503]. Additionally, a classification model based on 27 microbial species demonstrated accurate discrimination, with an area under the receiver operating characteristic curve of 0.84 and high disease specificity [504]. To predict immunotherapy responses, a supervised learning algorithm consistently forecasted outcomes of anti‐PD‐1 therapy and immune‐related adverse events across five cohorts [505]. A Phase II trial assessed the safety and tolerability of Neratinib, a tyrosine kinase inhibitor, in elderly patients with HER2^+^ breast cancer (NCT02673398). This study incorporated machine learning to develop a predictive model, successfully identifying microbial signatures linked to reduced drug toxicity [506]. Future research should focus on creating standardized clinical scoring systems that integrate microbiota, metabolic, and immune markers, thus advancing precision medicine [507]. To make machine learning for microbiome data analysis accessible to nonbioinformatics researchers, several software platforms have begun integrating machine learning modules [508, 509]. However, Gihawi et al. highlighted that some microbiome studies may contain data errors that affect the reliability of machine learning models [510].

The high heterogeneity of microbial composition among individuals, influenced by factors such as diet, geography, age, and medication, can limit the application of microbial biomarkers [511]. Furthermore, the lack of standardization in sampling methods, sequencing platforms, and data analysis processes restricts the comparability of studies and hampers clinical translation efficiency [512]. Environmental factors have been shown to dominate over host genetics in shaping the gut microbiota, creating substantial interindividual and interpopulation variability [513, 514]. These factors fundamentally undermine the applicability of universal microbiome‐based biomarkers across diverse clinical settings. Regional variation in gut microbiome composition alone is sufficient to confound disease‐associated microbial signatures, rendering reference ranges and machine learning classifiers trained in one population largely nontransferable to another [515]. Machine learning approaches for microbiome‐based prediction have demonstrated promise within single cohorts but remain vulnerable to these confounding factors and lack rigorous cross‐population validation [516, 517]. Patient stratification strategies, modeling by geographic origin, disease subtype, medication history, and age, offer a viable path forward by training population‐specific rather than universal classifiers. Despite these challenges, machine learning has demonstrated superior classification abilities compared with traditional methods, providing essential support for precise risk stratification [518]. Collectively, microbiota hold promising potential as a tool for early cancer diagnosis and prognosis prediction, but further clinical application still requires large‐scale prospective cohort validation.

## 12 Conclusions, Controversies, and Future Perspectives

The intricate relationship between the host microbiota and cancer represents one of the most dynamic frontiers in oncology. In this review, we have systematically dissected the host–microbiota–tumor axis, spanning tissue‐specific colonization patterns, molecular mechanisms of microbial signaling, immune and metabolic modulation, and emerging therapeutic strategies. The accumulating evidence firmly establishes that the microbiota is not merely a bystander but an active participant in cancer initiation, progression, and response to therapy. However, several critical challenges must be addressed before microbiota‐based approaches can be translated into routine clinical practice.

Although the study of the intratumoral microbiome holds great promise, the field faces significant rigor challenges due to the low‑biomass nature of samples. A notable example is an early study that claimed to identify distinct microbial signatures across multiple cancer types; upon reanalysis, its findings were invalidated due to major data‑processing errors, sequence misclassification, and uncontrolled batch effects and contamination [510]. This case underscores the common risks in the field and promotes a shift from mere sequencing associations toward multidimensional verification and causal validation. Given the high susceptibility of low‑biomass specimens to exogenous DNA contamination such as “kitome” and “splashome,” as well as technical noise introduced by batch effects across different extraction kits, sequencing platforms, and bioinformatics pipelines, amplicon sequencing alone is no longer sufficient to establish the presence of microbes within tumors [519, 520, 521]. Therefore, future studies must adopt more stringent strategies. On one hand, they should rigorously adhere to standardized protocols such as RIDE checklist and integrate multiple computational tools to systematically identify and remove contaminants [522, 523]. On the other hand, it is essential to move beyond mere sequencing correlations and actively incorporate orthogonal validation methods like FISH, CLEM, and culturomics [11, 195, 524].

Meanwhile, research on cancer‐associated microorganisms is currently influenced by significant variability in findings, which greatly undermines the reproducibility of results. This variability permeates the entire research process. During the preanalytical phase, differences in sample collection procedures and potential contamination introduce considerable batch effects. At the analysis stage, technical biases from sequencing platforms and the choice of reference databases can lead to substantial differences in classification results. Furthermore, publicly available datasets often lack adequate documentation or control for demographic factors such as race, geographic origin, and age, which may distort genuine biological signals [525, 526]. To improve the standardization of research, the STORMS guideline has been proposed to standardize reporting processes, enhancing the consistency and transparency of microbiome research [527].

Beyond these technical and analytical sources of variability, the relationship between gut microbiota and cancer development is further complicated by a range of host‐related confounding factors. Diet, lifestyle, medication use (particularly antibiotics and proton pump inhibitors), age, and geographic location all significantly shape gut microbial composition and may independently influence cancer risk. For instance, high‐fat diets increase the abundance of bile acid‐metabolizing bacteria, probably promoting inflammation‐associated carcinogenesis [528]. A fiber‐rich diet enriches SCFA‐producing bacteria with antitumor properties [529]. Chronic alcohol consumption changes intestinal permeability and microbial composition, facilitating bacterial translocation and systemic inflammation [530]. These confounders make it difficult to attribute cancer risk specifically to microbiota. Therefore, it is important to conduct well‐controlled prospective cohort studies with comprehensive dietary and lifestyle metadata to distinguish the causal contributions of the microbiota from the broader host and environmental influences.

Accounting for these confounders is a necessary step, yet an even deeper challenge lies in establishing causal relationships between specific microorganisms and cancers. For validating causality, the field has long relied on and continuously refined Koch's postulates. These traditional principles require that a pathogen is consistently present in patients, isolatable, capable of causing disease in a healthy host, and re‐isolatable from the newly infected host [531]. However, these postulates show inherent limitations when applied to complex, multifactorial diseases like cancer. For instance, many cancer‐associated microorganisms also colonize healthy individuals; some viruses cannot be cultured in pure form; and cancer development often involves multiple factors and long latency periods, making it difficult to faithfully replicate in animal models. Despite these challenges, researchers have successfully linked specific pathogens to cancers, such as H. pylori with GC, by flexibly adapting Koch's principles [532]. Yet, for most microorganisms, causal roles remain unproven. F. nucleatum, for example, is enriched in CRC but also commonly resides in the healthy oral cavity, and its mere introduction usually does not trigger tumorigenesis alone. This has accelerated the formation of more nuanced theoretical models. These include the “driver‐passenger model,” which distinguishes microorganisms that initiate cancer from those that proliferate afterward, and the “keystone pathogen hypothesis,” wherein certain low‐abundance bacteria may promote dysbiosis by disrupting immune balance [533, 534]. Even more complex is the “hit‐and‐run hypothesis,” which suggests microorganisms might initiate oncogenic onset early and then disappear from the tissue, evading detection in established cancers [535]. To address such complexity, modern research has developed multilevel causal inference frameworks. Molecular Koch's postulates shift the focus from whole microorganisms to specific virulence genes [536]. Ecological Koch's postulates consider “microbial dysbiosis” itself as a pathogenic entity [537]. Neville et al. proposed an inverse law for verifying the function of beneficial bacteria, aiming to find microorganisms that can prevent or treat diseases [538]. The prevailing consensus is that microorganisms are rarely sufficient as sole causes of cancers but instead act as a part of interactions involving host genetics and environmental exposures. Future causal studies are suggested to transcend cross‐sectional associations. It should pursue prospective longitudinal cohorts to determine whether microbial changes precede cancer development and actively design interventional experiments to verify functional roles

Although 11 microorganisms, including H. pylori, certain liver flukes (O. viverrini and C. sinensis), and oncogenic viruses such as HBV, HCV, and HPV, have been identified by the International Agency for Research on Cancer as direct carcinogens,, most microorganisms do not directly cause cancer but instead play a more complex role in tumor progression, typically promoting tumor development by regulating the host immune system [539]. Microbial communities within the TME have been detected in various cancers and are considered potential biomarkers for early diagnosis and prognosis prediction. For example, low‐biomass microbiota in blood or tissues can be amplified to predict cancer presence and stage, demonstrating high sensitivity and specificity [76].

Mechanistically, microbiota influence antitumor immunity through various pathways, including inflammatory signaling and SCFA metabolism, with particular emphasis on their role in the efficacy of ICIs. Microbiota‐based interventions, such as FMT, OVs, and engineered bacterial therapies, are emerging as novel therapeutic strategies. Among these, genetically engineered bacteria have been designed to release specific genes, drugs, and antibodies, thereby achieving localized activation of systemic anticancer immunity while minimizing systemic toxicity. However, the application of these strategies faces challenges, including selecting optimal donors, ensuring strain colonization efficiency, and avoiding potential pathogenic risks [540].

Future research is suggested to adopt multimodal integration that combines metagenomics with spatial transcriptomics and metabolomics at single‐cell resolution, enabling direct visualization of microbe–host interactions within the TME. Artificial intelligence and machine learning approaches will be helpful in revealing high‐dimensional microbiome datasets to identify robust biomarkers and predict therapeutic response. Mechanistic validation should advance beyond correlative studies through the use of tumor organoids, germ‐free animal models, and CRISPR‐based microbial engineering. Clinically, prospective trials with standardized protocols and comprehensive metadata collection are urgently needed to translate microbiota‐based diagnostics and therapeutics into practice.

## Author Contributions

L.J.L. and C.X. conceived, designed, and supervised the project. J.L., M.J.X, and L.Y conducted literature searches, drafted the manuscript, and prepared the figures. Z.Y.X. and Q.F.C. organized the tables. S.H.W. and T.K.W. organized references. L.J.L. and C.X. reviewed and edited the manuscript. All authors have read and approved the final version.

## Funding

This work was supported by the Noncommunicable Chronic Diseases‐National Science And Technology Major Project (2025ZD0543800), the Key Nature Science Foundation of Zhejiang Province (LZ24H160005), the Fundamental Research Funds for the Central Universities (226‐2025‐00249), the Fundamental Research Funds for the Central Universities (2025ZFJH03), the Central Guidance Fund for Local Science and Technology Development (2024ZY01054), and the Opening Foundation of the State Key Laboratory for Diagnosis and Treatment of Infectious Diseases, The First Affiliated Hospital, Zhejiang University School of Medicine (SKLID2025KF01).

## Ethics Statement

The authors have nothing to report.

## Conflicts of Interest

The authors declare no conflicts of interest.

## Data Availability

No datasets were generated or analyzed during the current study.
